# Supplementary material for: A scoping review of the use of traditional medicine for the management of ailments in West Africa
Source: PLoS One. 2024 Jul 8;19(7):e0306594. doi: 10.1371/journal.pone.0306594 (PMC11230574; doi:10.1371/journal.pone.0306594)
Supplement: S1 File — (DOCX) [file pone.0306594.s002.docx]

**Supplemental File 1: Search Strategy for the CINAHL, Web of Science and Pubmed**

Total:6948

Duplicate:540

CINAHL complete=70

| S/N | Keywords | Search Terms |
| --- | --- | --- |
| 1 | Traditional medicine | TI (traditional medicine or complementary and alternative medicine or herb medicine) OR TI (herbal medicine or herbalism or herbal supplements) OR TI medicinal plant extracts OR TI african traditional medicine OR TI indigenous medicine OR TI Ethnomedicine |
| 2 | Africa | TI Africa OR TI (Saharan africa or sub-saharan africa or Sahara or sub-sahara or ssa) |
|  |  |  |

Web of Science= 2768

| S/N | Keywords | Search Terms |
| --- | --- | --- |
| 1 | Traditional medicine | **((((((((TS=(Traditional medicine )) OR TS=(Herbal medicine )) OR TS=(Medicinal plant)) OR TS=(Plant extract)) OR TS=(Herbal drugs)) OR TS=(African Traditional medicine )) OR TS=(Indigenous Medicine)) OR TS=(Home Remedies)) OR TS=(Ethnomedicine)** and **Preprint Citation Index** (Exclude – Database) |
| 2 | West Africa | **((((((((((((((((TS=(west africa)) OR TS=(Benin)) OR TS=(Burkina Faso)) OR TS=(Cape Verde)) OR TS=(Côte D'Ivoire)) OR TS=(Gambia)) OR TS=(Ghana)) OR TS=(Guinea)) OR TS=(Guinea-Bissau)) OR TS=(Liberia)) OR TS=(Mali)) OR TS=(Mauritania)) OR TS=(Niger)) OR TS=(Nigeria)) OR TS=(Senegal)) OR TS=(Sierra Leone)) OR TS=(Togo)** and **Preprint Citation Index** (Exclude – Database) |

PubMed= 646

|  | Keywords | Search strategy |
| --- | --- | --- |
| #1 | Traditional Medicine | **((((((((Herbal medicine[Title/Abstract]) OR (Traditional medicine[Title/Abstract])) OR (Medicinal plant[Title/Abstract])) OR (Plant extract[Title/Abstract])) OR (Herbal drugs[Title/Abstract])) OR (African Traditional medicine[Title/Abstract])) OR (Indigenous Medicine[Title/Abstract])) OR (Home Remedies[Title/Abstract])) OR (Ethnomedicine[Title/Abstract])** |
| #2 | Use | “use"[Title/Abstract] |
| #3 | Africa | “Africa"[Title/Abstract] |
| #4 | #1 AND #2 AND #3 |  |
