# Supplementary material for: A scoping review of the use of traditional medicine for the management of ailments in West Africa
Source: PLoS One. 2024 Jul 8;19(7):e0306594. doi: 10.1371/journal.pone.0306594 (PMC11230574; doi:10.1371/journal.pone.0306594)
Supplement: S2 File — (DOCX) [file pone.0306594.s003.docx]

**Supplemental file 2: A compendium of plant families used for medicinal purposes in West Africa**

1. Alliaceae [60]
2. Acanthaceae [24, 27, 36, 39, 45, 48, 61]
3. Aizoaceae [15]
4. Aloaceae [27, 60]
5. Amaranthaceae [16, 24, 27, 32, 39, 60, 62]
6. Amaryllidaceae [39, 62]
7. Anacardiaceae [24, 32, 39, 58 60-62]
8. Anisophylleaceae [58]
9. Annonaceae [27, 39, 45, 58]
10. Anonaceae [32, 60, 62]
11. Apiaceae [15]
12. Apocynaceae [27, 39, 45, 58, 60- 62]
13. Araceae [32, 39]
14. Araliaceae [39]
15. Arecaceae [15, 24, 39, 58, 60]
16. Asclepiadaceae [15, 277, 32, 36, 60]
17. Asparagaceae [58]
18. Asteraceae [24, 27, 36, 39, 45, 58, 60, 61]
19. Balanitaceae [15]
20. Basellaceae [24]
21. Bignoniaceae [16, 27, 36, 58, 60, 62]
22. Bombacaceae [15, 27, 60]
23. Boraginaceae [27, 39, 47, 60, 62]
24. Bromeliaceae [39]
25. Burseraceae [15, 39, 60-62]
26. Caesalpiniaceae [32, 47]
27. Calophyllaceae [39]
28. Cannabaceae [58]
29. Capparaceae [15, 58, 60, 62]
30. Capparidaceae [27]
31. Caricaceae [32, 39, 58, 61, 62]
32. Caryophyllaceae [60, 61]
33. Casuarinaceae [39]
34. Celastraceae [15, 32, 39, 58, 60]
35. Chrysobalanaceae [27, 58]
36. Cleomaceae [39]
37. Clusiaceae [39, 61, 62]
38. Cochlospermaceae [47]
39. Combretaceae [15, 27, 37, 39, 58, 60, 62]
40. Compositae [32, 62]
41. Connaraceae [32, 39, 58, 62]
42. Convolculaceae [32, 56, 60]
43. Crassulaceae [24, 32, 39]
44. Crassulaceae [39]
45. Cucurbetaciae [15, 27, 36, 39, 45, 59, 60, 62]
46. Cyperaceae [15, 27, 39, 58, 60]
47. Dennstaedtiaceae [27]
48. Dichapetalaceae [45, 62]
49. Dilleniaceae [58]
50. Dioscoreaceae [36, 39, 58, 60]
51. Ditleniaceae [32]
52. Ebenaceae [32, 58, 60]
53. Euphorbiaceae [16, 24, 27,32, 39, 45, 58, 60-62]
54. Fabaceae [15, 16, 24, 27, 39, 58]
55. Flacourtiaceae [32]
56. Gentianaceae [58]
57. Guttiferae [61]
58. Hypericaceae [32, 58]
59. Hypoxidaceae [58, 60, 62]
60. Icacinaceae [58, 62]
61. Iridaceae [62]
62. Irvingiaceae [24],
63. Labiatae [32, 36]
64. Lamiaceae [24, 27, 39, 58, 60-62]
65. Lauraceae [58]
66. Leguminosae [36, 37, 45, 47, 60, 62]
67. Liliaceae [16, 45]
68. Loganiaceae [16, 32, 47, 58, 60, 61]
69. Loranthaceae [36, 45, 58]
70. Lythraceae [62]
71. Malpighiaceae [58]
72. Malvaceae [15, 24, 27, 32, 39, 58, 60, 62]
73. Marantaceae [39, 62]
74. Melastomataceae [32, 58, 60, 61]
75. Meliaceae [15, 16, 27, 32, 39, 45, 47, 58, 60, 62]
76. Menispermaceae [16, 27, 39, 58,61]
77. Mimosaceae [32]
78. Moraceae [24, 32, 60]
79. Moraceae [39, 58]
80. Moringaceae [27, 58, 60]
81. Musaceae [39, 60, 62]
82. Myristicaceae [16, 39]
83. Myrtaceae [16, 27, 39, 45, 60, 62]
84. Nyctaginaceae [16, 24, 39]
85. Nymphaeaceae [15, 60],
86. Olacaceae [37, 45, 58, 62]
87. Onagraceae [60]
88. Opiliaceae [37, 47, 58]
89. Orchidaceae [58]
90. Oxalidaceae [37]
91. Palmae [32, 36]
92. Pandaceae [38]
93. Papaveraceae [36, 39, 58]
94. Papilionaeeae [32, 47]
95. Passifloraceae [16, 32, 39, 58, 61]
96. Pedaliaceae [32]
97. Phyllanthaceae [39, 58, 62]
98. Piperaceae [36, 39, 58, 61, 62]
99. Plantaginaceae [58]
100. Poaceae [15, 36, 39, 58, 60, 62]
101. Polygalaceae [39, 58, 60, 62]
102. Portulaceae [32, 39, 60]
103. Primulaceae [60]
104. Rhamnaceae [15 32]
105. Rhizophoraceae [45],
106. Rubiaceae [15, 16, 32, 39, 45, 58, 61, 62]
107. Rutaceae [24, 27, 32, 39, 45, 58, 60-62]
108. Salicaceae [39, 62]
109. Salvadoraceae [15]
110. Sapindaceae [24, 27, 36, 39, 58, 60, 62]
111. Sapotaceae [24, 27, 36, 58, 60]
112. Selaginellaceae [32]
113. Simaroubaceae [58]
114. Smilacaceae [58, 60]
115. Solanaceae [24, 27, 32, 36, 39, 45, 58, 60, 62]
116. Sterculiaceae [15, 37, 60]
117. Talinaceae [24]
118. Thymelaeaceae [58]
119. Tiliaceae [15, 36]
120. Verbenaceae [16, 39, 58, 60- 62]
121. Vitaceae [58, 60, 61]
122. Zingiberaceae [16, 32, 36, 39, 45, 58, 60-62]
123. Zygophyllaceae [39, 45, 60, 62]
