# Supplementary material for: A scoping review of the use of traditional medicine for the management of ailments in West Africa
Source: PLoS One. 2024 Jul 8;19(7):e0306594. doi: 10.1371/journal.pone.0306594 (PMC11230574; doi:10.1371/journal.pone.0306594)
Supplement: S3 File — (DOCX) [file pone.0306594.s004.docx]

**Supplemental file 3: Compendium of plant families used for experimentation**

1. Acanthaceae [45]
2. Agavaceae [44]
3. Amaranthaceae [40, 44. 29, 35]
4. Annonaceae [29, 34, 35, 44, 55, 56]
5. Apocynaceae [28, 40, 44, 55, 59]
6. Araliaceae [44]
7. Asclepiadaceae [40]
8. Asparagaceae [44]
9. Aspergillaceae [49]
10. Asteraceae [40]
11. Astraceae [46, 59]
12. Bignoniaceae [44, 59]
13. Bombacaceae [40, 46, 59]
14. Caesalpinaceae [44]
15. Celastraceae [44]
16. Chrysobalanaceae [44]
17. Cochlospermaceae [44]
18. Combretaceae [14, 23, 30, 35, 44, 51, 55]
19. Commelinaceae [46, 39]
20. Convolvulaceae [44]
21. Costaceae [56]
22. Cyperaceae [44]
23. Daisy [25, 35, 52]
24. Davalliaceae [46, 59]
25. Euphorbiaceae [18, 21, 29, 38, 39, 41, 42, 44, 45, 47, 50, 55],
26. Fabaceae [29, 35, 44]
27. Hymenocardiaceae [44]
28. Hyppocrateaceae [44]
29. Lamiaceae [17]
30. Legumes [50]
31. Leguminoceae [45, 53]
32. Loganiaceae [44]
33. Loranthaceae [45]
34. Lythraceae [55]
35. Madder [35]
36. Mahogany [22]
37. Mallows [50]
38. Malvacae [44, 46, 59]
39. Meliaceae [44, 55]
40. Mimosaceae [40, 44]
41. Moraceae [44, 46, 59]
42. Moringaceae [29]
43. Olacaceae [44, 45]
44. Opiliaceae [44, 55]
45. Orchidaceae [59]
46. Papilionaceae [55]
47. Passifloraceae [55]
48. Piperaceae [30]
49. Polygalaceae [44]
50. Rhizophoraceae [45]
51. Rubiaceae [19, 20, 26, 30, 35, 40, 44, 46, 55, 57, 59]
52. Rutaceae [30, 33, 35, 45, 46, 59]
53. Sapindaceae [46, 55, 59]
54. Sapotaceae [30, 35]
55. Simarubaceae [46, 59]
56. Solanacea [45, 50]
57. Sterculiaceae [44]
58. Verbenaceae [44, 55, 59]
59. Vitaceae [44]
60. Zingiberaceae [45, 54, 55]
