# Supplementary material for: A scoping review of the use of traditional medicine for the management of ailments in West Africa
Source: PLoS One. 2024 Jul 8;19(7):e0306594. doi: 10.1371/journal.pone.0306594 (PMC11230574; doi:10.1371/journal.pone.0306594)
Supplement: Attachment — Submitted filename: PONE-D-23-43939.pdf [file pone.0306594.s006.pdf]

PLOS ONE

A scoping review on the use of traditional medicine for the management of ailments in West Africa

--Manuscript Draft--

**Manuscript Number:**	PONE-D-23-43939
**Article Type:**	Research Article
**Full Title:**	A scoping review on the use of traditional medicine for the management of ailments in West Africa
**Short Title:**	Traditional medicines and health in West Africa
**Corresponding Author:**	Morẹ́nikẹ́ Oluwátóyìn Foláyan, FWACS<br>Obafemi Awolowo University<br>Ile-Ife, Osun State NIGERIA
**Keywords:**	Complimentary medicines; Alternative medicines; Herbal remedy; Africa
**Abstract:**	Background<br><br>The coexistence of traditional healing practices deeply rooted in cultural and historical contexts and the evolving landscape of modern healthcare approaches in West African societies creates a dynamic interplay between tradition and modernity in healthcare. This study aims to comprehensively explore the landscape of traditional medicine use for health in West Africa, considering diverse ethnicities, languages, and cultural traditions.<br><br>Methods<br><br>A scoping review was conducted following the Joanna Briggs Institute (JBI) methodology and reported according to the Preferred Reporting Items for Systematic Reviews and Meta-Analyses Extension for Scoping Reviews (PRISMA-ScR) guidelines. Research questions focused on the links between traditional medicine practices and health in West Africa. The systematic literature search covered PubMed, Web of Science, and CINAHL from database inception to September 2023. Descriptive analysis was conducted highlighting the years of publication, countries of publication, study designs plant families and plant parts used for making traditional medicines, and the diseases the traditional remedies are made for.<br><br>Results<br><br>The search identified 3484 records, with 46 articles meeting inclusion criteria. Publications spanned from 1979 to 2023, revealing an increasing interest in traditional medicine. Nigeria had the highest number of publications (54.3%), followed by Ghana (19.6%). The studies employed various designs, including experimental, ethnobotanical, ethnopharmacological, and clinical trials. Plant families frequently studied included Euphorbiaceae, Rubiaceae, and Combretaceae. Traditional remedies addressed a wide range of health issues, from general symptoms to specific diseases, highlighting their versatility.<br><br>Conclusion<br><br>This scoping review provides a comprehensive overview of traditional healing practices in West Africa. The identified studies underscore the need for culturally sensitive healthcare interventions. The prevalence of traditional medicine use and the diversity of practices emphasize the importance of fostering collaborative efforts between traditional healers and modern healthcare professionals. The study identifies gaps in knowledge and areas for future research, laying the groundwork for further investigations into the nuanced healthcare ecosystem of West Africa.
**Order of Authors:**	Morẹ́nikẹ́ Oluwátóyìn Foláyan, FWACS
	Selassi A D’Almeida

Powered by Editorial Manager® and ProduXion Manager® from Aries Systems Corporation

	Sahr E Gbomor
	Brima Osaio-Kamara
	Mobolaji Timothy Olagunju
	Olunike Rebecca Abodunrin
**Additional Information:**	
**Question**	**Response**
**Financial Disclosure**<br><br>Enter a financial disclosure statement that describes the sources of funding for the work included in this submission. Review the submission guidelines for detailed requirements. View published research articles from *PLOS ONE* for specific examples.<br><br>This statement is required for submission and **will appear in the published article** if the submission is accepted. Please make sure it is accurate.<br><br>**Funded studies**<br>Enter a statement with the following details:<br>• Initials of the authors who received each award<br>• Grant numbers awarded to each author<br>• The full name of each funder<br>• URL of each funder website<br>• Did the sponsors or funders play any role in the study design, data collection and analysis, decision to publish, or preparation of the manuscript?<br><br>Did you receive funding for this work?	The author(s) received no specific funding for this work.
**Competing Interests**<br><br>Use the instructions below to enter a competing interest statement for this submission. On behalf of all authors, disclose any competing interests that could be perceived to bias this work—acknowledging all financial support and any other relevant financial or non-financial competing interests.	The authors have declared that no competing interests exist

This statement is required for submission and **will appear in the published article** if the submission is accepted. Please make sure it is accurate and that any funding sources listed in your Funding Information later in the submission form are also declared in your Financial Disclosure statement.<br><br>View published research articles from *PLOS ONE* for specific examples.<br><br>**NO authors have competing interests**<br><br>Enter: *The authors have declared that no competing interests exist.*<br><br>**Authors with competing interests**<br><br>Enter competing interest details beginning with this statement:<br><br>*I have read the journal's policy and the authors of this manuscript have the following competing interests: [insert competing interests here]*<br><br>* typeset	
**Ethics Statement**<br><br>Enter an ethics statement for this submission. This statement is required if the study involved:<br><br>• Human participants<br>• Human specimens or tissue<br>• Vertebrate animals or cephalopods<br>• Vertebrate embryos or tissues<br>• Field research<br><br>Write "N/A" if the submission does not require an ethics statement.<br><br>General guidance is provided below. Consult the submission guidelines for detailed instructions. **Make sure that all information entered here is included in the Methods section of the manuscript.**	N/A

Powered by Editorial Manager® and ProduXion Manager® from Aries Systems Corporation

**Format for specific study types**<br><br>**Human Subject Research (involving human participants and/or tissue)**<br>• Give the name of the institutional review board or ethics committee that approved the study<br>• Include the approval number and/or a statement indicating approval of this research<br>• Indicate the form of consent obtained (written/oral) or the reason that consent was not obtained (e.g. the data were analyzed anonymously)<br><br>**Animal Research (involving vertebrate animals, embryos or tissues)**<br>• Provide the name of the Institutional Animal Care and Use Committee (IACUC) or other relevant ethics board that reviewed the study protocol, and indicate whether they approved this research or granted a formal waiver of ethical approval<br>• Include an approval number if one was obtained<br>• If the study involved *non-human primates*, add *additional details* about animal welfare and steps taken to ameliorate suffering<br>• If anesthesia, euthanasia, or any kind of animal sacrifice is part of the study, include briefly which substances and/or methods were applied<br><br>**Field Research**<br><br>Include the following details if this study involves the collection of plant, animal, or other materials from a natural setting:<br>• Field permit number<br>• Name of the institution or relevant body that granted permission	
**Data Availability**<br><br>Authors are required to make all data underlying the findings described fully available, without restriction, and from the time of publication. PLOS allows rare exceptions to address legal and ethical concerns. See the PLOS Data Policy and FAQ for detailed information.	Yes - all data are fully available without restriction

A Data Availability Statement describing where the data can be found is required at submission. Your answers to this question constitute the Data Availability Statement and **will be published in the article**, if accepted.<br><br>**Important:** Stating 'data available on request from the author' is not sufficient. If your data are only available upon request, select 'No' for the first question and explain your exceptional situation in the text box.<br><br>Do the authors confirm that all data underlying the findings described in their manuscript are fully available without restriction?	
**Describe where the data may be found in full sentences. If you are copying our sample text, replace any instances of XXX with the appropriate details.**<br><br>• If the data are **held or will be held in a public repository**, include URLs, accession numbers or DOIs. If this information will only be available after acceptance, indicate this by ticking the box below. For example: *All XXX files are available from the XXX database (accession number(s) XXX, XXX.*).<br>• If the data are all contained **within the manuscript and/or Supporting Information files**, enter the following: *All relevant data are within the manuscript and its Supporting Information files.*<br>• If neither of these applies but you are able to provide **details of access elsewhere**, with or without limitations, please do so. For example:<br><br>*Data cannot be shared publicly because of [XXX]. Data are available from the XXX Institutional Data Access / Ethics Committee (contact via XXX) for researchers who meet the criteria for access to confidential data.*<br><br>*The data underlying the results presented in the study are available*	All relevant data are within the manuscript and its Supporting Information files.

from (include the name of the third party and contact information or URL).<br>• This text is appropriate if the data are owned by a third party and authors do not have permission to share the data.<br><br>* typeset	
Additional data availability information:	

Manuscript

Click here to access/download;Manuscript;Traditional medicines for health care in West Africa_Final.docx

A scoping review on the use of traditional medicine for the management of ailments in West Africa

Selassi A D'Almeida, Sahr E Gbomor, Brima Osaio-Kamara, Mobolaji Timothy Olagunju[4] Olunike Rebecca Abodunrin,[4] Moréniké Oluwátóyìn Foláyan,[5,6]

1. Universal Health Coverage Life Course Cluster , World Health Organisation, Freetown, Sierra Leone
2. Department of Complementary and Alternative Medicine, Pharmacy Board of Sierra Leone, Freetown, Sierra Leone
3. Directorate of Primary Health Care, Ministry of Health, Freetown, Sierra Leone
4. Department of Epidemiology and Biostatistics, Nanjing Medical University, Jiangsu Province, China
5. Department of Child Dental Health, Obafemi Awolowo University, Ile-Ife
6. Oral Health Initiative, Nigerian Institute of Medical Research, Yaba, Lagos, Nigeria

Corresponding author

Morenike Oluwatoyin Folayan

Department of Child Dental Health

Obafemi Awolowo University, Ile-Ife

toyinukpong@yahoo.co.uk

// Abstract

Background

The coexistence of traditional healing practices deeply rooted in cultural and historical contexts and the evolving landscape of modern healthcare approaches in West African societies creates a dynamic interplay between tradition and modernity in healthcare. This study aims to comprehensively map the landscape of traditional medicine use for health in West Africa.

Methods

A scoping review was conducted following the Joanna Briggs Institute (JBI) methodology and reported according to the Preferred Reporting Items for Systematic Reviews and Meta-Analyses Extension for Scoping Reviews (PRISMA-ScR) guidelines. Research questions focused on the links between traditional medicine practices and health in West Africa. The systematic literature search covered PubMed, Web of Science, and CINAHL from database inception to September 2023. Descriptive analysis was conducted highlighting the years of publication, countries of publication, study designs plant families and plant parts used for making traditional medicines, and the diseases the traditional remedies are made for.

Results

The search identified 3484 records, with 46 articles meeting inclusion criteria. Publications spanned from 1979 to 2023, revealing an increasing interest in traditional medicine. Nigeria had the highest number of publications (54.3%), followed by Ghana (19.6%). The studies employed various designs, including experimental, ethnobotanical, ethnopharmacological, and clinical trials. Plant families frequently studied included Euphorbiaceae, Rubiaceae, and Combretaceae. Traditional remedies addressed a wide range of health issues, from general symptoms to specific diseases, highlighting their versatility.

Conclusion

This scoping review provides a comprehensive overview of traditional healing practices in West Africa. The identified studies underscore the need for culturally sensitive healthcare interventions. The prevalence of traditional medicine use and the diversity of practices emphasize the importance of fostering collaborative efforts between traditional healers and modern healthcare professionals. The study identifies gaps in knowledge and areas for future research, laying the groundwork for further investigations into the nuanced healthcare ecosystem of West Africa.

Key words: Complimentary medicines; Alternative medicines; Herbal remedy; Africa

Introduction

The dynamic interplay between tradition and modernity in healthcare practices remains a complex and intriguing facet of West African societies [1]. This interplay reflects the ongoing negotiation between longstanding traditional healing methods deeply rooted in cultural and historical contexts and the evolving landscape of modern healthcare approaches [2]. In West Africa, traditional healing practices often involve indigenous knowledge, rituals, and the use of natural remedies passed down through generations [3]. These practices coexist with, and at times, intersect with modern medical interventions, technologies, and pharmaceuticals thereby reflecting the adaptability and resilience of West African societies in navigating the complexities of health and well-being [4]. Patients often engage with both traditional and modern healthcare systems, sometimes concurrently or sequentially, depending on the nature of their health needs, personal beliefs, and accessibility [5]. This health-seeking behaviors across the region creating a nuanced healthcare ecosystem [6] and underscores the importance of acknowledging diverse healthcare practices and fostering collaborative efforts between traditional healers and modern healthcare professionals [7, 8].

As global interest in alternative and complementary medicine grows [9], understanding the prevalence and scope of traditional healing practices in West Africa becomes paramount for fostering culturally sensitive and effective healthcare interventions. It is therefore important to gather and synthesize information to provide a comprehensive overview of traditional healing practices in West Africa. This will help identify key themes, patterns, and trends in the literature related to traditional healing practices, thereby allowing for nuance understanding of the prevalence and variations of these practices across different regions, communities, and cultural contexts in West Africa. By systematically reviewing the existing literature, gaps in knowledge

and areas where further research is needed can be identified, and future studies and interventions needed can be identified.

This scoping review tried to comprehensively explore the landscape of traditional medicine use for health in West Africa, a region characterized by diverse ethnicities, languages, and cultural traditions, exhibits a rich tapestry of indigenous healing practices [10]. The review systematically maps out the various modalities employed in traditional medicine and discusses the implications of traditional medicine for public health in West Africa.

Methods

The methodology was based on the Joanna Briggs Institute (JBI) scoping review methodology [11] and reported following the Preferred Reporting Items for Systematic Reviews and Meta-Analyses Extension for Scoping Reviews guidelines (PRISMA-ScR) [12,13].

Identifying research questions

The review was guided by the research questions: what are the published links between traditional medicine practices and health in West Africa?

Identifying relevant studies

A systematic search of the literature was conducted on PubMed, Web of Science, and the Cumulative Index of Nursing and Allied Health Literature (CINAHL) using the terms shown in Appendix 1. The search was conducted from the inception of the database till September 2023. A search of related citations and references was also carried out.

Study selection

Publications identified through the search strategy were downloaded into Endnote, and imported into Rayyan, and duplicates were removed. Three researchers (ORA, MTO, MOF) performed a screening of the titles and abstracts of the downloaded articles independently using pre-defined inclusion and exclusion criteria. Studies were included if there was an agreement between all the reviewers. Disparities were settled by consensus. In addition, the three researchers completed the full-text review (ORA, MTO, MOF). Uncertainty regarding whether publications met the inclusion criteria was resolved via consensus among the three researchers.

Inclusion criteria

Peer-reviewed journal articles, books, book chapters, conference proceedings, and reports that cover traditional medicine and health focusing on West Africa. Studies included were published in the English Language, addressed both preventive and curative aspects of oral and explored herbal remedies, rituals, or cultural beliefs. Studies reporting outcomes related to the impact of traditional medicine on preventive and curative health, including remedies used during epidemics and pandemics, were included. There was no restriction on study design or date of publication.

Exclusion criteria

Animal studies were excluded. Also excluded were non-African populations unless they provide crucial comparative insights, unpublished theses and dissertations, letters to the editor, commentaries on studies, scoping, systematic and narrative reviews, and studies whose full lengths cannot be accessed were excluded. Also, studies with insufficient data or methodology not suitable for analysis, and those published on websites were also excluded.

Data charting process

A data-charting form was developed to extract relevant variables. The charted variables were the literature characteristics (e.g., authors, year of publication), study aim, study design, form of traditional medicines used, diseases managed, and the outcomes of the studies.

Data analysis

The results of the scoping review were reported according to the PRISMA-ScR checklist. A deductive analysis was conducted using the framework developed for the data extraction. Details generated from the analysis were the years of publication, regions in Africa where studies were conducted, forms of traditional medicines used (plants, animals, fungi, or geological elements), diseases (and diseases pathogenic organisms) investigated, plants (and plant extracts) investigated, and forms and routes of administration of the traditional medicines for the management of diseases. Descriptive analysis was conducted highlighting the years of publication, countries of publication, study designs plant families and plant parts used for making traditional medicines, and the diseases the traditional remedies are made for.

Results

The search resulted in the identification of 3484 records, which were downloaded into Endnote and imported into Rayyan. After de-duplication, 3395 records remained. After reviewing titles and abstracts, screening, 226 articles were eligible for full-text screening. On screening the full articles, 13 articles were excluded either because full articles were not available, the focus of the study was not Africa or the study was not related to health, leaving 46 articles [14-59] for this review. Figure 1 shows the flow diagram of the publication screening process.

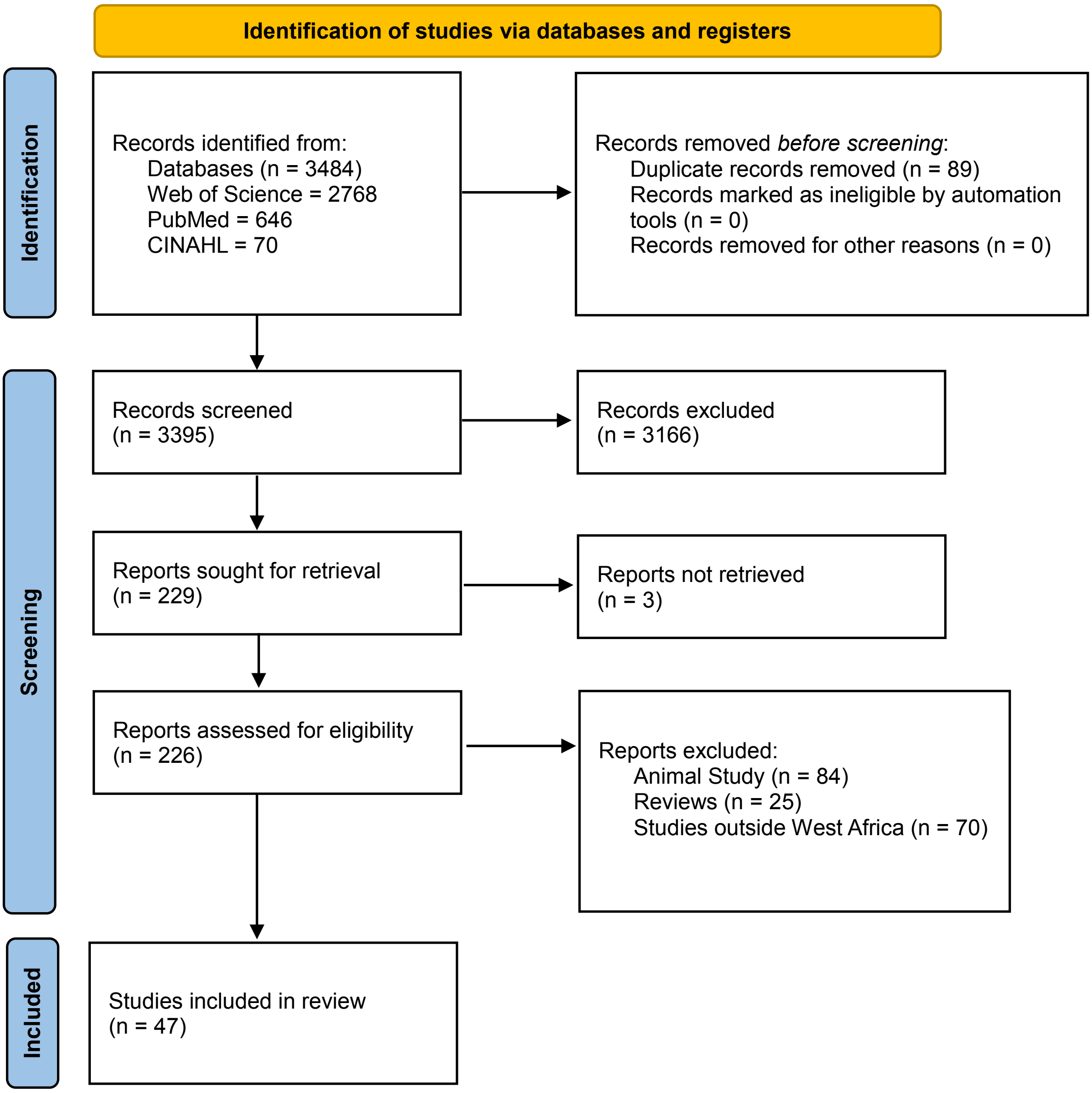

From: Page MJ, McKenzie JE, Bossuyt PM, Boutron I, Hoffmann TC, Mulrow CD, et al. The PRISMA 2020 statement: an updated guideline for reporting systematic reviews. BMJ 2021;372:n71. doi: 10.1136/bmj.n71

As shown in Table 1, the publication period for the 46 manuscripts spanned from 1979 to 2023. The distribution across time revealed four (8.7%) were published between 1979 and 1990 [31-33, 35], 20 (43.5%) were published between 1991 and 2000 [14-19, 20-23, 25, 38, 41-43, 46, 49, 51, 57, 59], 13 (28.3%) were from 2001 to 2010 [26, 36, 37, 40, 44, 47, 48, 50, 52-56], six (13.0%) papers from 2011 to 2020 [27-30, 39, 58], and three (6.5%) papers from 2021 to 2023 [24, 34, 45].

Among the 17 West African countries, only eight countries have publications on traditional medicines. These countries include Nigeria, which accounts for 25 (54.3%) of the publications [14, 16-18, 20-26, 30, 31, 33-36, 38, 41-43, 48, 49, 53, 56], Ghana accounting for nine (19.6%) of the publications [27-29, 39, 40, 45, 50, 52, 54], Mali [15, 37, 47, 57] and Togo [19, 46, 51, 59] accounting for four (8.7%) publications each, Cote d'Ivoire accounting for two (4.3%) publications [44, 55], and Sierra Leone [32], Benin [51], and Guinea Bissau [58], each having one (2.2%) publication. There was a publication produced from Togo and Benin [51].

The studies encompassed various types of study design ranging from experimental studies conducted in laboratories to determine the bioactivities of identified traditional remedies [14, 17-19, 21-23, 25, 26, 28-31, 33-35, 38, 40-43, 44, 46, 49, 51-54, 56, 57, 59]; to ethnobotanical studies which are studies focus on the traditional knowledge, practices, and uses of plants by local communities [15, 16, 20, 24, 27, 32, 36, 45, 58]; ethnopharmacological investigations which are the investigation of traditional knowledge related to the medicinal uses of plants, animals, and other natural substances within specific cultural or ethnic groups [37, 39, 47, 55]; and clinical trials which are systematic investigations conducted on human participants to evaluate the safety, efficacy, and effects of medical treatments, interventions, or therapies [48, 50].

Table 1: Characteristics of published manuscripts on traditional medicines in West Africa

S/no	Title	Author (Year of publication)	Country	Study Aim	Study design
1	Anti-Sickling Potential of Terminalia catappa Leaf Extract	Mgbemene et al., 1999 [14]	Nigeria	To investigate the potential of Terminalia catappa leaves in the management of sickle cell disorders by inhibiting hemolysis and preventing sickling of erythrocytes.	Experimental study
2	An Ethnobotanical Survey of Herbal Drugs of Gourma District, Mali	Diallo et al., 1999 [15]	Mali	To analyze the knowledge and use of medicinal plants in the Gourma region of Mali and identify potential sources for new bioactive compounds in Malian medicine	Ethnobotanical survey
3	Herbs for mental disorders	Nwosu, 1999 [16]	Nigeria	To report on the traditional use of herbal remedies in southern Nigeria for the management of mental disorders	Ethnobotanical survey
4	Antidiarrhoeal Activities of Ocimum gratissimum (Lamiaceae)	Ilori. 1996 [17]	Nigeria	To investigate the antidiarrhoeal activities of leaf extracts of Ocimum gratissimum	Experimental study
5	Antimicrobial activities of crude leaf extracts of wilkesiana Acalypha	Alade, 1993 [18]	Nigeria	To investigate the antimicrobial activities of crude leaf extracts of Acalypha wilkesiana	Experimental study
6	Effects of Three Compounds Extracted from Morinda lucida on Plasmodium falciparum	Koumaglo, 1992 [19]	Togo	To investigate the effects of three compounds extracted from Morinda lucida on the growth of Plasmodium falciparum in vitro	Experimental Study
7	Antifungal activities of crude extracts of Mitracarpus villosus (Rubiaceae)	Irobi, 1993 [20]	Nigeria	To investigate the antifungal activities of crude extracts from Mitracarpus villosus	Experimental study
8	Effects of crude leaf extracts of Acalypha torta against some anaerobic bacteria	Irobi, 1994 [21]	Nigeria	To analyze the antibacterial activities of crude leaf extracts of Acalypha torta against certain anaerobic bacteria	Experimental study
9	Anti-malarial activity of Nigerian neem leaves	Udeinya, 1993 [22]	Nigeria	To investigate the anti-malarial activity of neem leaves extract	Experimental study
10	Antibacterial activity of some medicinal plants from Nigeria	Olukoya, 1993 [23]	Nigeria	To investigate the antibacterial activity of ten medicinal plants used for the treatment of various microbial infections in Nigeria	Experimental study

11	Ethnobotanical use-pattern for indigenous fruits and vegetables among selected communities in Ondo State, Nigeria	Olowo, 2022 [24]	Nigeria	To document the ethnobotanical use-pattern of indigenous fruits and vegetables in selected communities in Ondo State, Nigeria	Ethnobotanical survey
12	Activities of *Chromolaena odorata* (Compositae) leaf extract against *Pseudomonas aeruginosa* and *Streptococcus faecalis*	Irobi, 1992 [25]	Nigeria	To investigate the antibacterial activities of Chromolaena odorata leaf extract against Pseudomonas aeruginosa and Streptococcus faecalis	Experimental study
13	Screening of the leaves of three Nigerian medicinal plants for antibacterial activity	Adomi, 2008 [26]	Nigeria	To screen the leaves of three Nigerian medicinal plants for antibacterial activity	Experimental Study
14	Documentation of Herbal Medicines Used for the Treatment and Management of Human Diseases by Some Communities in Southern Ghana	Boadu and Asase, 2017 [27]	Ghana	To document herbal medicines used by traditional healers to treat and manage human diseases and ailments in southern Ghana.	Ethnobotanical Study
15	Activity of Herbal Medicines on Plasmodium falciparum Gametocytes: Implications for Malaria Transmission in Ghana	Amoah et al, 2015 [28]	Ghana	To assess the efficacy and gametocydal effects of commonly used herbal malaria products in Ghana and their implications for malaria transmission.	Experimental Study
16	In vitro Antiplasmodial Activities of Aqueous Extracts of Selected Ghanaian Herbal Plants on the Development of Asexual and Sexual Stage Malaria Parasites	Cudjoe et al, 2020 [29]	Ghana	To examine the in vitro antiplasmodial activities of aqueous extracts of selected Ghanaian herbal plants on the development of asexual and sexual stage malaria parasites.	Experimental study
17	In vitro assessment of aqueous and ethanolic extracts of some Nigerian chewing sticks on bacteria associated with dental infections	David et al, 2010 [30]	Nigeria	To investigate the antimicrobial efficacy of aqueous and ethanolic extracts of ten Nigerian chewing sticks on bacteria associated with dental infections	Experimental study
18	Traditional medicine and lead-containing preparations in Nigeria	Healy et al, 1984 [31]	Nigeria	To examine the use of heavy metals, especially lead, in traditional medicines and cosmetics employed by the Nigerian communities in Britain	Experimental Study

19	Medicinal plants in Pujehun District of Sierra Leone	MacFoy and Sama, 1983 [32]	Sierra Leone	To document the medicinal plants used by the local people in Pujehun District for the treatment of various diseases and ailments	Ethnobotanical survey
20	Antimicrobial Alkaloids from a Nigerian Chewing Stick (Fagara zanthoxyloides)	Odebiyi and Sofowora, 1979 [33]	Nigeria	To investigate the antimicrobial properties of alkaloids extracted from a Nigerian chewing stick, Fagara zanthoxyloide	Experimental Study
21	In vitro antiviral activity of twenty-seven medicinal plant extracts from Southwest Nigeria against three serotypes of echoviruses	Ogbole et al, 2021 [34]	Nigeria	To evaluate the antiviral activity of 27 medicinal plant extracts against three serotypes of echoviruses (E6, E7, and E11) that cause various human diseases	Experimental study
22	The effect of crude extracts of nine African chewing sticks on oral anaerobes	Rotimi and Mosadomi, 1986 [35]	Nigeria	To test the in vitro susceptibility of four species of oral anaerobic bacteria to crude extracts from nine popular chewing sticks used for dental and oral hygiene in Nigeria	Experimental study
23	Use of medicinal plants for the treatment of measles in Nigeria	Sonibare et al, 2009 [36]	Nigeria	To conduct an ethnobotanical survey of three Local Government areas of the Ijebu area of Ogun State in southwest Nigeria for plants used in the treatment of measles.	Ethnobotanical survey
24	Ethnopharmacological Survey of Six Medicinal Plants from Mali, West-Africa	Grønhaug et al., 2008 [37]	Mali	To collect information about the use of six medicinal plants in the regions around Siby and Dioila, Mali	Ethnopharmacological Survey
25	Antimicrobial Constituents of the Leaves of *Acalypha wilkesiana* and *Acalypha hispida*	Adesina et al., 2000 [38]	Nigeria	To isolate and identify the antimicrobial principles of *A. Wilkesiana* and *A. Hispida.*	Experimental study
26	An ethnopharmacological survey of medicinal plants traditionally used for cancer treatment in the Ashanti region, Ghana.	Agyare et al., 2018 [39]	Ghana	To find out how traditional healers in the Ashanti region of Ghana recognize and classify cancer, how they treat the disease with medicinal plants especially in children, and to investigate the collection, identification, preparation, and application of these plants for cancer treatment.	Ethnopharmacological survey
27	Herbal Remedies Traditionally Used Against Malaria in Ghana: Bioassay-Guided Fractionation of *Microglossa pyrifolia* (Asteraceae)	Köhler et al., 2002 [40]	Ghana	To investigate the antiplasmodial activity of 11 plants from Ghana, where malaria causes many deaths per year, particularly in young children, and to choose 1 of them,	Experimental study

				Microglossa pyrifolia, for further investigation.	
28	Antibacterial activity of *Alchornea cordifolia* stem bark	Ajali, 2000 [41]	Nigeria	To report the preliminary results of antimicrobial screening of the extracts of Alchornea cordifolia stem bark	Experimental study
29	The Antimicrobial Activity of Roots of *Jatropha podagrica* (Hook)	Aiyelaagbe et al., 2000 [42]	Nigeria	To explore the possible antimicrobial agents in the roots of *Jatropha podagrica* and to identify the active compounds	Experimental study
30	Antibacterial activity of *Piliostigma thonningii* stem bark	Akinpelu & Obuotor. 2000 [43]	Nigeria	To determine the antibacterial activity of *Piliostigma thonningii* stem bark extract	Experimental study
31	Traditional medicine in North Côte-d'Ivoire: screening of 50 medicinal plants for antibacterial activity	Koné et al., 2004 [44]	Côte-d'Ivoire	To screen 50 medicinal plants used in North Côte d'Ivoire as traditional remedies for bacterial diseases for in vitro antibacterial activity against Gram-negative and Gram-positive bacteria.	Experimental study
32	Ethnobotanical Survey and Cercaricidal Activity Screening of Medicinal Plants Used for Schistosomiasis Treatment in Atwima-Nwabiagya District, Ashanti Region, Ghana	Asante-Kwatia et al., 2023 [45]	Ghana	To highlight the importance of medicinal plants in the treatment of schistosomiasis in Ghanaian communities and to investigate their anti-schistosomal activity	Ethnobotanical survey
33	Further investigations on the antiviral activities of medicinal plants of Togo	Hudson et al., 2000 [46]	Togo	To determine antiviral activities of 10 species of Togolese medicinal plants, previously shown to possess activity against herpes simplex virus	Experimental study
34	Ethnopharmacological survey of different uses of seven medicinal plants from Mali, (West Africa) in the regions Doila, Kolokani and Siby	Togola et al., 2005 [47]	Mali	To identify different uses of certain medicinal plants in Mali and investigate their uses in traditional medicine	Ethnopharmacological survey
35	Clinical Evaluation of Acalypha Ointment in the Treatment of Superficial Fungal Skin Diseases	Oyelami et al., 2003 [48]	Nigeria	To evaluate the efficacy and safety of Acalypha wilkesiana ointment in superficial fungal skin diseases	Open non-comparative clinical trial

36	Antibiotic activity of *Aspergillus quadrilineatus* extracts isolated from a Nigerian cereal	Irobi et al., 2000 [49]	Nigeria	To determine the time-course for the induction of antibiotic activity and the effects of different carbon sources, pH, temperature and bivalent cations on the antibiotic activity of *Aspergillus quadrilineatus* extracts	Experimental study
37	Evaluation of Efficacy and Safety of a Herbal Medicine Used for the Treatment of Malaria	Ankrah et al., 2003 [50]	Ghana	To determine the efficacy and safety of a decoction, AM-1 used to treat malaria at a herbal clinic in Ghana	Clinical trial and experimental study
38	Antifungal activities of seven West African *Combretaceae* used in traditional medicine	Baba-Mousa et al., 1999 [51]	Benin and Togo	To investigate the antifungal properties of 7 species of the *Combretaceae* family that are abundant in West Africa.	Experimental study
39	Antimicrobial activity of the leaves and flowering tops of *Acanthospermum hispidum*.	Fleischer et al., 2002 [52]	Ghana	To investigate the antimicrobial activity of the leaves and flowering tops of *Acanthospermum hispidum*	Experimental study
40	Antimicrobial activity of *Pterocarpu*s *osun* stems	Ebi and Ofoefule, 2003 [53]	Nigeria	To examine the antimicrobial properties of *Pterocarpus osun* stems.	Experimental study
41	Antimicrobial activity of some medicinal plants from Ghana.	Konning et al., 2003 [54]	Ghana	The report the results of a preliminary antimicrobial screening of the methanol extracts of Aframomum melegueta, Piper guineense, Xylopia aethiopica, Zingiber officinale, medicinal plants of Ghana	Experimental study
42	Antiparasitic activities of medicinal plants used in Ivory Coast	Okpekon et al., 2004 [55]	Cote d'Ivoire	To evaluate in vitro crude extracts of these plants in a drug screening against malaria, leishmaniasis, African trypanosomiasis, helminths and scabies	Experimental study
43	The leaf essential oil of *Costus afer* Ker-Grawl from Nigeria	Taiwo and Bolanle, 2003 [56]	Nigeria	The absence of reports on the leaf essential oil composition of this plant in the literature prompted this Investigation.	Experimental study
44	Antimalarial Activity of Four Plants used in Traditional Medicine in Mali.	Traore-Keita et al., 2000 [57]	Mali	To evaluate in vitro the antimalarial activity and the cytotoxicity to macrophages of 4 plants used in traditional medicine for treating periodic fevers	Experimental study

45	Medicinal plants of Guinea-Bissau: Therapeutic applications, ethnic diversity and knowledge transfer	Catarino et al., 2016 [58]	Guinea-Bissau	To prepare a comprehensive documentation of Guinea Bissau's medicinal plants.	Ethnobotanical survey
46	Investigation of Medicinal Plants of Togo for Antiviral and Antimicrobial Activities	Anani et al., 2000 [59]	Togo	To determine the antiviral and antibiotic activities of 19 medicinal plants of Togo.	Experimental study

Table 2 highlights the characteristics of the 11 ethnobotanical and ethnopharmacological surveys included in the scoping review. The studies were conducted in Nigeria (n=4), Ghana (n=3), Mali (n=2), Sierra Leone (n=1) and Guinea-Bissau (N=1).

A hundred and sixteen plant families were identified for use as medicinal plants. Appendix 2 provides a compendium of these plant. The most cited plant family was Meliaceae [15, 16, 27, 32, 39, 45, 47, 58].

The part of the plants used for medicinal purposes range from the aerial part of plants [58] to barks [15, 16, 24, 27, 37, 39, 45, 47, 58], branches [32, 58], climbers [39], flowers [39, 58], fruits [15, 24, 27, 39, 45, 47], gum [15], leaves [15, 16, 24, 27, 32, 36, 37, 45, 47, 48, 58], male plant-inflorescences [16], pods [16, 37], rhizome [15, 16, 39], roots [15, 27, 47, 24, 32, 36, 37, 39, 45, 58], sap [39, 58], seeds [15, 16, 24, 36, 39, 45, 58], stems [15, 16, 24, 32, 39, 58], thorns [15], tubers [39], twigs [16, 36, 37], and whole plants [15, 27, 39, 45, 58].

The prepared remedies could be applied topically applied [15, 36, 39], inhaled [16], or intranasally applied [15, 39]. It can be prepared as a decoction [15, 16, 27, 32, 36, 37, 39, 47], powder [15, 37, 39, 47], poultice [15, 32, 39], oil [39], juice [15, 32, 36, 39], macerated [15, 39, 47] or taken as food [24, 58], soup [36, 39], tea [39] or taken as raw fruit [39]. The remedies could also be used for bathing [36], as an enema [15], for infusion [16, 27, 32, 47], as a fumigant [15], or for gargling [15, 32, 37], shampoo [15].

The remedies were used to manage general symptoms such as aches and pains [15, 24, 32, 37, 47, 58], diarrhea [15, 24, 32, 37], odema, wounds and burns [15, 24, 32, 37, 47, 58], dizziness [15, 24], convulsion [24, 27, 32], jaundice [15, 27, 37, 47], bites and sting [24, 27, 37, 58] to

specific diseases like malaria/fever [15, 24, 27, 32, 37, 47, 58], measles [24, 32, 36], diabetes [24, 27, 58], sexually transmitted diseases [24, 32, 37, 58], yellow fever [24, 32, 37], leprosy cholera [24], [24, 37], sinusitis [15], cancer [24, 39] tuberculosis [24], and schistosomiasis [32, 45, 47]. Other disorders managed are anemia and blood disorders [24, 27, 32, 58], cold, cough and respiratory diseases [15, 24, 32, 37, 58], diseases of the eyes [15, 24, 32, 47, 58], diseases of the kidney [37, 58], diseases of the liver [24, 47, 58], gastrointestinal problem [15, 24, 27, 32, 37, 47, 58], skin problems [15, 24 27, 32, 37, 47, 58], haemorrhoid [15, 27, 32, 47, 58], mental and neurological disorders [16, 24, 58], neuromuscular problems [15, 24, 27, 47, 58], gynaecological problems [24, 27, 32, 37, 47, 58], cardiovascular problems [24, 27, 32, 47], child health problems [27, 32, 47, 58], oral health problems [15, 24, 27, 32, 37], and hernia [27, 58]. The remedies are also used as anthelmintic [15, 24, 37], antiseptic [15], diuretic [27], bone fracture [24], for the management of poisoning [32, 58], baldness [27], dropsy [37], foot rot [27], and to prevent frequent urination when drunk [32].

Table 2. Characteristics of the ethnobotanical and ethnopharmacological surveys included in the scoping review

S/no	Author (Year of publication)	Country	Forms in which products are used	Part of plan made from	Diseases plants are used to manage	Family of plants used for disease management
1	Diallo et al., 1999 [14]	Nigeria	Decoction, powder, boiled fruit added to meals, maceration, shampoo, juice, gargling, fumigation, enema, topical application, intranasal application, poultice,	Stems, leaves, fruit, root, root bark, thorns, gum, whole plant, rhizome, seeds	Abdominal pain, jaundice, dizziness, stimulate appetite, malaria, against heart disease, cold, nausea, vomiting, diarrhoea, dysentery, chest pain, inflammations, wound management, sinusitis, dyspepsia, haemorrhoid, constipation, tonsillitis, gum inflammation; conjunctivitis, asthma, expel roundworms, toothache, antiseptic, oedema, spots and pimples, headache, flatulence, pneumomia, boils, sprain, body pain, laxative	Aizoaceae, Arecaceae, Apiaceae, Asclepiadaceae, Balanitaceae, Bombacaceae, Burseraceae, Capparaceae, Celastraceae, Combretaceae, Cucurbetaciae, Cyperaceae, Fabaceae, Malvaceae, Meliaceae, Nymphaeaceae, Rhamnaceae, Poaceae, Rubiaceae, Salvadoraceae, Sterculiaceae, Tiliaceae
2	Nwosu, 1999 [16]	Nigeria	Decoctions, aqueous extracts, infusions, stem exudate, inhalation	Leave, twig, stem bark, root bark, seed, male plant-inflorescences, ginger rhizomes, pod	Mental disorders including amnesia, insomnia, senile dementia, depression, delusion of persecution, delusion of grandeur, schizophrenia, and psychosomatic disorders	Amaranthaceae, Liliaceae, Nyctaginaceae, Rubiaceae, Fabaceae, Euphorbiaceae, Meliaceae, Menispermaceae, Verbenaceae, Zingiberaceae, Myristicaceae, Bignoniaceae, Passifloraceae, Myrtaceae, Loganiaceae.
3	Olowu, 2022 [24]	Nigeria	Not stated	Leaves, fruits, bark, seeds, stems, roots, whole plant, stem,	Anaemia, arthritis, asthma, blood tonic, body pains, boils, bone fractures, boosts libido, cancer, cholera, cleanses liver, colds, conjunctivitis, constipation, convulsions, coughs, diabetes, diarrhoea, dizziness, dog bites, dysentery, earache, eczema, immune booster, enhances lactation, epilepsy, fertility, fever, fibroids, gonorrhoea, headache, heart attack, hypertension, improves digestion, increases blood flow, inflammation, laxative, leprosy, madness, malaria, measles, pneumonia, promotes labour, rheumatism, ringworm, scabies, scorpion bites, skin infection, snake bites, sores, toothache, tumours, tuberculosis.,	Asteraceae, Acanthaceae, Moraceae, Basellaceae, Sapindaceae, Nyctaginaceae, Crassulaceae, Malvaceae, Amaranthaceae, Sapotaceae, Rutaceae. Lamiaceae, Euphorbiaceae, Arecaceae. Fabaceae, Irvingiaceae, Solanaceae, Anacardiaceae, Talinaceae

					typhoid, ulcer, vomiting, wound healing, yellow fever, hepatitis, good eyesight.	
4	Boadu and Asase, 2017 [27]	Ghana	Decoctions, infusions.	Leaves, whole plant, root, bark, fruit	High blood pressure, stops bleeding, piles, diabetes, malaria, typhoid fever, convulsion, menstrual disorders, stroke, halitosis, baldness, Heart burns, sexual disorders, osteoarthritis, diuretic, stomach ulcer, low sperm count, Snake bite, infertility, hernia, miscarriage, foot rot, retarded growth, rheumatism, jaundice, Pruritus, blood tonic.	Acanthaceae, Aloaceae, Amaranthaceae, Annonaceae, Apocynaceae, Asclepiadaceae, Asteraceae, Bignoniaceae, Bombacaceae, Boraginaceae, Capparidaceae, Chrysobalanaceae, Combretaceae, Cucurbitaceae, Cyperaceae, Dennstaedtiaceae, Euphorbiaceae, Fabaceae, Lamiaceae, Malvaceae, Meliaceae, Menispermaceae, Moringaceae, Myrtaceae, Rutaceae, Sapindaceae, Sapotaceae, Solanaceae.
5	MacFoy and Sama, 1983 [32]	Sierra Leone	Decoction, infusion, poultice, juice or gargled	Leaves, roots, branches, stem	Wounds, eases labour pains, stomach pain, reduce swollen stomach, abdominal pains, gonorrhoea, bilharzia, poison of the stomach caused by witches, skin inflammation, yellow fever, laxative, malaria, cough, palpitation of the heart, toothache, gum pain, haemostasis, dysentery, bilharzia, diarrhea, convulsion, closure of the frontal suture in babies, heals inflammation, prevents frequent urination when drunk, eye troubles, haemorrhoids, causes abortion during early pregnancy, vaginal rash, eruptive skin disease, boil, measles, asthma, promotes closure of the frontal suture in babies, controls persistent menstruation.	Amaranthaceae, Anacardiaceae, Anonaceae, Araceae, Asclepiadacae, Caesalpiniaceae, Caricaceae, Celastraceae, Compositae, Convolculaceae, Connaraceae, Crassulaceae, Ditleniaceae, Ebenaceae, , Euphorbiaceae, Flacourtiaceae, Hypericaceae, Labiatae, Loganiaceae, Malvaceae, Melastomataceae, Meliaceae, Mimosaceae, Moraceae, Palmae, Papilionaceae, Passifloraceae, Pedaliaceae, Portulaceae, Rhamnaceae, Rubiaceae, Rutaceae, Selaginellaceae, Solanaceae, Zingiberaceae
6	Sonibare, 2009 [36]	Nigeria	Taken as soup, juice of the fresh leaves, decoctions, ointment, mixed into soap for bathing	Twigs, roots, seeds, leaves	Measles	Acanthaceae, Asclepiadaceae, Asteraceae, Bignoniaceae, Cucurbitaceae, Dioscoreaceae, Labiatae, Leguminosae, Loranthaceae, Palmae, Papaveraceae, Piperaceae, Poaceae, Sapindaceae, Sapotaceae, Solanaceae, Tiliaceae, Zingiberaceae
7	Grønhaug et al., 2008 [37]	Mali	Decoction or powder Gum used as a gargle.	Leaves, roots, stem bark, twig, pod	Pain, fever, diarrhoea, blennorrhoe, wound, chest-affections, constipation, leprosy, sore throat, dysentery, dropsy,	*Biopyhtum petersianum* (Oxalidaceae), *Cola cordifolia* (Sterculiaceae), *Combretum mole* (Combretaceae), *Opilia celtidifolia*

					ascites and oedemas, jaundice, yellow fever, dermatitis, malaria, stomachache, purgative gonorrhoea, kidney stone, dental abscess, headache, anthelmintic, dysmenorrhea, antidote against scorpion sting and snake bite	(Opiliaceae), *Parkia biglobosa* (Leguminosae) and *Ximenia americana* (Olacaceae).
8	Agyare et al., 2018 [39]	Ghana	Poultice, oil, decoction, raw fruit, exudate, maceration (topical), tea, juice massage, soup, squeezed fruit for drink, powder (topical/oral), cream, nasal drop, tincture	Roots, bark, leaves, fruit, climbers, stems, rhizome, sap, tuber, seed, whole plant, flower	Prostrate, stomach, breast, brain, lungs, skin, cervical, ovarian, genital, bone, joint, heart, and throat cancer	Acanthaceae, Amaranthaceae, Amaryllidaceae, Anacardiaceae, Annonaceae, Apocynaceae, Araceae, Araliaceae, Arecaceae, Asteraceae, Boraginaceae, Bromeliaceae, Burseraceae, Calophyllaceae, Caricaceae, Casuarinaceae, Celastraceae, Cleomaceae, Clusiaceae, Combretaceae, Connaraceae, Crassulaceae, Crassulaceae, Cucurbitaceae, Cyperaceae, Dioscoreaceae, Euphorbiaceae, Fabaceae, Lamiaceae, Malvaceae, Marantaceae, Meliaceae, Menispermaceae, Moraceae, Musaceae, Myristicaceae, Myrtaceae, Nyctaginaceae, Pandaceae, Papaveraceae, Passifloraceae, Phyllanthaceae, Piperaceae, Poaceae, Polygalaceae, Portulaceae, Rubiaceae, Rutaceae, Salicaceae, Sapindaceae, Solanaceae, Verbenaceae, Zingiberaceae, Zygophyllaceae
9	Asante-Kwatia et al., 2023 [45]	Ghana	Not stated	Leaves, whole plant, roots, stem bark, seed, fruit	Schistosomiasis	Asteraceae, Euphorbiaceae, Liliaceae, Apocynaceae, Rhizophoraceae, Meliaceae, Zygophyllaceae, Zingiberaceae, Dichapetalaceae, Acanthaceae, Loranthaceae, Cucurbitaceae, Rubiaceae, Myrtaceae, Solanaceae, Olacaceae, Leguminoseae, Annonaceae, Rutaceae
10	Togola et al., 2005 [47]	Mali	Decoction, infusion of powder and maceration	Leaves, stem bark, root, fruits.	Malaria, abdominal pain, dermatitis, amenorrhoea, urinary bilharzias, sterility, baby thinness, ocular infections, high blood pressure, hepatitis, jaundice, chest pain, arthritis, headache, wound healing, haemorrhoids, backache, abdominal flatulence, child malnutrition	Opiliaceae, Loganiaceae, Papilionoideae, Boraginaceae, Meliaceae, Leguminosae, Caesalpinioideae, Cochlospermaceae.

11	Catarino et al., 2016 [58]	Guinea-Bissau	Food	Flowers, stem, roots, whole plants, branches, bark, leaves, stem, seeds, sap, aerial part of plants	Health problems managed using the plants are pains, pregnancy, childbirth, breastfeeding and diseases of the newborn related problems, gastrointestinal problem, skin inflammations, wounds and burns, cough and respiratory diseases, malaria/fever, stings, bites and poisoning, mental and neurological disorders, sexually transmitted diseases, anemia and blood disorders, male impotence, diseases of the eyes, diseases of the liver, diseases of the kidney, rheumatism and arthritis, hemorrhoids, heart conditions, bones and joints, neuromuscular problems, inguinal hernia, diabetes, and also some unspecified health conditions.	Acanthaceae, Amaranthaceae, Anacardiaceae, Annonaceae, Anisophylleaceae, Apocynaceae, Arecaceae, Asteraceae, Asparagaceae, Bignoniaceae, Cannabaceae, Capparaceae, Caricaceae, Celastraceae, Chrysobalanaceae, Combretaceae, Connaraceae, Convolvulaceae, Cucurbitaceae, Cyperaceae, Dilleniaceae, Dioscoreaceae, Ebenaceae, Euphorbiaceae, Fabaceae, Gentianaceae, Hypericaceae, Hypoxidaceae, Icacinaceae, Lamiaceae, Lauraceae, Loganiaceae, Loranthaceae, Malpighiaceae, Malvaceae, Melastomataceae, Meliaceae, Menispermaceae, Moraceae, Moringaceae, Olacaceae, Opiliaceae, Orchidaceae, Papaveraceae, Passifloraceae, Phyllanthaceae, Piperaceae, Plantaginaceae, Poaceae, Polygalaceae, Rubiaceae, Rutaceae, Sapindaceae, Sapotaceae, Simaroubaceae, Smilacaceae, Solanaceae, Thymelaeaceae, Verbenaceae, Vitaceae, Zingiberaceae

Table 3 highlights the characteristics of the 36 experimental studies and clinical trials included in the scoping review. These studies were conducted in Nigeria (n=22), Ghana (n=7), Togo (n=4), Cote d'Ivoire (n=2), Republic of Benin (n=1), Mali (n=1). A single study was conducted in both Togo and the Republic of Benin [51].

The effect of medicinal plants were explored against the following group of diseases and disorders: sickle cell [14], gastrointestinal disorders including diarrhea and dysentery [17, 23, 31, 34, 54], respiratory disorders like cough [23, 34, 43, 54, 56], malaria/fever [19, 22, 28, 29, 40, 41, 50, 55-57], dental infection [30, 33, 35], skin infections [20, 23, 31, 44, 54, 55] and wound infections [21, 43, 53]. Other disorders managed were eye infection [31, 56], infertility [31], leprosy [41, 43], ulcers [43], meningitis/encephalitis [34], smallpox [43], urinary tract infection [44, 56], sore throat [23], gonorrheal [23], schistosomiasis [45] and rhematic pain specifically [41, 54, 56]. Some studies explored specific antifungal [18, 20, 42, 48, 49, 51], antibacterial [18, 20-23, 25, 26, 30, 35, 42, 46, 49, 52, 53, 59] and antiviral [34, 46, 59] effects of plant extracts.

Sixty plant families were studied (See Appendix 3 for the compendium). The most common plant family studied were Euphorbiaceae [18, 21, 29, 38, 40, 41, 42, 44, 45, 47, 50, 55], Rubiaceae [19, 20, 26, 30, 35, 40, 44, 46, 55, 57, 59] and Combretaceae [14, 23, 30, 35, 44, 51, 55]. The 12 studies on Euphorbiacea were conducted in Nigeria [18, 21, 38, 41, 42, 48] and Ghana [29, 40, 45, 50] and Cote D'Ivoire [44, 55]. The plant family studied in the most diverse country was the Rubiaceae studied in Togo [19, 46, 59], Nigeria [20, 26, 30, 35], Ghana [40], Cote D-Ivoire [44, 55] and Mali [57]. Combretaceae was studied in Nigeria [14, 23, 30, 35], Cote D'Ivoire [44, 55], Togo [51] and Republic of Benin [51].

Plant families reported in studies conducted in Nigeria include Lamiaceae [17], Euphorbiacea [18, 21, 38, 41, 41, 48], Rubiaceae [20, 26, 30, 35], Mahogany [20], Combretaceae [14, 23, 30, 35], Daisy [25, 35], Annonaceae [34], Sapotaceae [30, 35], Fabaceae [30, 35], Piperaceae [30], Rutaceae [30, 33, 35], Anacardiaceae [35], Aspergillaceae [49], Leguminosea [53], Costaceae [56], Rutaceae [35] and Madder [35].

Plant families reported in studies conducted in Ghana include Euphorbiacea [29, 40, 45, 50], Daisy [52], Apocynaceae [28, 40], Annonaceae [29, 35], Moringaceae [29], Anacardiaceae [29], Zingiberaceae [54], Amaranthaceae [40] Asclepiadaceae [40], Asteraceae [40], Bombacaceae [40], Mimosaceae [40], Rubiaceae [40], Mallows [50], Solanaceae [45, 50], Legumes [50], Olacaceae [45], Acanthaceae [45], Zingiberaceae [45], Leguminoceae [45], Rhizophoraceae [45], Rutaceae [45], and Loranthaceae [45].

Plant families reported in studies conducted in Togo include Rubiaceae [19, 46], Combretaceae [51], Malvacae [46, 59], Astraceae [46, 59], Commelinaceae [46, 59], Bombacaceae [46, 59], Davalliaceae [46, 59], Moraceae [46, 59], Rutaceae [46, 59], Simarubaceae [46, 59], Sapindaceae [46, 59], Apocynaceae [59], Bignoniaceae [59], Orchidaceae [59], Rubiaceae [59], Verbenaceae [59].

Plant families reported in studies conducted in Cote d'Ivoire include Combretaceae [44, 55], Annonnaceae [44, 55], Lythraceae [55]. Agavaceae [44], Amaranthaceae [44], Anacardiaceae [44], Apocynaceae [44, 55], Araliaceae [44], Asparagaceae [44], Bignoniaceae [44], Caesalpinaceae [44], Celastraceae [44], Chrysobalanaceae [44], Cochlospermaceae [44], Convolvulaceae [44], Cyperaceae [44], Euphorbiaceae [44, 55], Fabaceae [44], Hymenocardiaceae [44], Hyppocrateaceae [44], Loganiaceae [44], Malvaceae [44], Meliaceae

[44, 55], Mimosaceae [44], Moraceae [44], Olacaceae [44], Opiliaceae [44, 55], Polygalaceae [44], Rubiaceae [44, 55], Sterculiaceae [44], Verbenaceae [44, 55], Vitaceae [44], Combretaceae [55], Papilionaceae [55], Passifloraceae [55], Sapindaceae [55], and Zingiberaceae [55].

The plant family reported in Benin was Combretacea [51]. The plant family studied in Mali was Rubiaceae [57].

Table 3: Characteristics of the experimental studies and clinical trials included in the scoping review

S/no	Author (Year of publication)	Country	Form of traditional medicine (plant family)	Diseases explored	Study findings
1	Mgbemene et al., 1999 [14]	Nigeria	*Terminalia catappa* leaf ethanol extract (Combretaceae)	Sickle cell disorder	Extract inhibited osmotically induced hemolysis of human erythrocytes in a dose-dependent manner. It also prolonged the clotting time of uncoagulated blood. Additionally, a 1.0 mg/ml solution of the extract was effective in preventing and reversing the sickling of human 'SS' erythrocytes induced by a sodium metabisulphite solution.
2	Ilori, 1996 [17]	Nigeria	Extracts of *Ocimum gratissimum* leaf (Lamiaceae)	Diarrhoeal diseases	The extracts were active against *Aeromonas sobria, Escherichia coli, Plesiomonas shigelloses, Salmonella typhi*, and *Shigella dysenteriae*. but most active against *S. dysenteriae* and least active against *Salmonella typhi*.
3	Alade, 1993 [18]	Nigeria	Water and ethanol extracts of leaves of *Acalypha wilkesiana* (Euphorbiaceae)	Antimicrobial activities of extracts against bacteria and fungi.	The extracts inhibited the growth of bacteria and fungi. The aqueous extract was found to be static in action, while the ethanolic extract was uniformly tidal in effect.
4	Koumaglo, 1998 [19]	Togo	Decoctions of the aerial part, stem bark, or root bark of *Morinda lucida* (Rubiaceae)	Malaria and other tropical diseases	The three compounds extracted showed dose-dependent inhibition of the growth of Plasmodium falciparum in vitro.
5	Irobi, 1993 [20]	Nigeria	Ethanolic extracts of *Mitracarpus villosus* (Rubiaceae)	Skin infections caused by *Trichophyton rubrum, Microsporum* gypseum, *Candida albicans, Aspergillus niger, and Fusarium solani*	Extracts showed definite antifungal activities against the tested fungi. Aqueous extracts and the glycerol vehicle control did not inhibit any of the fungi. The zones of inhibition produced by the ethanol extracts ranged from 10 to 20.5 mm. The minimum inhibitory concentration of the extracts ranged from 0.50 to 4.0 mg/ml, and their minimum fungicidal concentration values ranged from 1 to 8 mg/ml. The extracts were fungistatic at lower concentrations and fungicidal at higher concentrations.
6	Irobi, 1994 [21]	Nigeria	*Acalypha torta* extracts (Euphorbiaceae)	Anaerobic bacteria associated with wound infections.	The aqueous, ethanol, and methanol extracts of A. torta showed in vitro antibacterial activities against all the anaerobic bacteria tested. The extracts exerted bactericidal effects on the bacteria at larger doses.
7	Udeinya, 1993 [22]	Nigeria	Neem leaves extract (Mahogany)	Malaria	Neem leaves extract exhibited significant anti-malarial activity, leading to the complete cessation of parasite development.

8	Olukoya, 1993 [23]	Nigeria	Water and ethanol extracts from species of Combretaceae	Gonorrhea, sore throat, diarrhea, abscess, boils, dysentery, coughs, body sores	Several of the tested plants exhibited antimicrobial activity against a range of microorganisms, including bacteria such as Streptococcus, Neisseria gonorrhoeae, Salmonella, Shigella, Escherichia coli, and more
9	Irobi, 1992 [25]	Nigeria	Ethanolic leaf extract of *Chromolaena odorata* (Daisy)	Pseudomonas aeruginosa and Streptococcus faecalis.	The ethanolic leaf extract of Chromolaena odorata exhibited antibacterial activity against both Pseudomonas aeruginosa and Streptococcus faecalis. The extract was more effective against Streptococcus faecalis than Pseudomonas aeruginosa.
10	Adomi, 2008 [26]	Nigeria	Aqueous and ethanol extracts of the leaves of *Morinda lucida* (Rubiaceae)	Antibacterial activity	Morinda lucida extract was active against all tested bacteria, while the latex of *Alstonia boonei* showed no activity against any of the bacteria tested.
11	Amoah et al, 2015 [28]	Ghana	Aqueous and ethanol extracts of *Cryptolepis sanguinolenta* (Apocynaceae) and *Azadirachta indica (*Meliaceae)	Malaria	Some herbal antimalarial products showed potential gametocydal activities, which could reduce the transmission of malaria parasites.
12	Cudjoe et al, 2020 [29]	Ghana	Extract from *Alchornea cordifolia(*Euphorbiaceae)*, Polyalthia longifolia* (Annonaceae)*, Moringa oleifera (*Moringaceae)*,* and *Mangifera indica (*Anacardiaceae)	Malaria	Alchornea cordifolia exhibited activity against the three tested parasites and effectively cleared both asexual parasites and gametocytes. Moringa oleifera and Mangifera indica also showed antimalarial activities. The study also identified the presence of phenolic flavonoids and other phytochemicals in the herbal extracts, which have been associated with antimalarial activity.
13	David, 2010 [30]	Nigeria	Chewing sticks made from Sapotaceae, Fabaceae, Combretaceae, Piperaceae, Rutaceae, Meliaceae, Clusiaceae, and Rubiaceae	Bacteria associated with dental infections	The ethanolic extracts had higher antimicrobial activity compared to the aqueous extracts. Viellaria paradoxical showed a zone of inhibition ranging between 5 and 13 mm, with Moraxella catarrhalis being the most susceptible bacterium to the extracts. Actinomyces vercosus was resistant to the extracts of Piper guineense, Azadirachta indica, and Massularia accumunata.
14	Healy et al, 1984 [31]	Nigeria	Tiro	Eye cleaner	The samples of tiro collected from Babalawo (native doctor), Eleweomo (herbalist) and homemade products in both the two and village contained and average of 50.1% (w/w) of lead.
15	Odebiyi and Sofowora, 1979 [33]	Nigeria	Ethanolic extract of the root of *Fagara zanthoxyloides* (Rutaceae)	Toothaches	The extract yielded four pure compounds which showed antimicrobial activity. One of these compounds is a tertiary phenolic alkaloid (carithine–6–one), two are quarternary alkaloids (chelerythrine chloride and berberine chloride),

					while the structure of the fourth compound was not determined.
16	Ogbole et al, 2021 [34]	Nigeria	Methanol extracts of dried Annonaceae	Echovirus infections causing meningitis, encephalitis, respiratory illness, gastroenteritis	Out of the 27 extracts tested, 11 showed antiviral activity against at least one echovirus serotype. The extracts had low cytotoxicity, indicating their safety for use.
17	Rotimi and Mosadomi, 1986 [35]	Nigeria	Crude aqueous or alcoholic extracts from the stems and or roots of *Serindeia warneckei* (Anacardiaceae), *Fagara zanthoxyloides* (Rutaceae), *Distemonanthus benthamianus* (Fabaceae), *Massularia accuminata* (Rubiaceae), *Anogeissus leiocarpus*(Combretaceae), *Vernonia amygdalina (*Daisy), *Bytyrospermum paradoxum* (Sapotaceae), *Terminalia glaucescens* (Combretaceae), *Nauclea latifolia* (Madder)	Dental caries, periodontal disease, and other oral diseases	All the extracts showed inhibitory effects on the growth of the oral anaerobes, with varying degrees of potency. *Serindeia warneckei* had the greatest and most consistent inhibitory effect on the four species studied namely *Bacteroides oralis, Bacteroides melaninogenicus, Bacteroides gingivalis, Bacteroides asaccharolyticus*
18	Adesina et al., 2000 [38]	Nigeria	Aqueous ethanol extract of *Acalypha wilkesiana* and *Acalypha hispida* leaves (Euphorbiaceae)	Antimicrobial activity of the leaves.	Gallic acid, corilagin, and geraniin were identified as the compounds responsible for the observed antimicrobial activity in the leaves of *Acalypha wilkesiana* and *Acalypha hispida.*
19	Köhler et al., 2002 [40]	Ghana	Aqueous leaf decoction, leaf extract, and infusion of *Gomphrena celosioides* (Amaranthaceae), *Picralima nitida* (Apocynaceae), *Pergularia daemia* (Asclepiadaceae), *Emilia sonchifolia* (Asteraceae), *Microglossa pyrifolia* (Asteraceae), *Adansonia digitata* (Bombacaceae), *Euphorbia hirta, Phyllanthus niruroides* (Euphorbiaceae), *Tetrapleura tetraptera* (Mimosaceae), *Mitragyna inermis, Mitragyna stipulosa* (Rubiaceae)	Fever and malaria	Different extracts from the plants showed promising in vitro activity against *Plasmodium falciparum*.
20	Ajali, 2000 [41]	Nigeria	Stem bark of *Alchornea cordifolia* (Euphorbiaceae)	Fever, rheumatic pains, purgative, and leprosy	The extracts of *Alchornea cordifolia* stem bark exhibited antimicrobial activities, supporting some uses of the plant in traditional medicine.

21	Aiyelaagbe et al., 2000 [42]	Nigeria	Stem and roots of *Jatropha podagrica* as chewing sticks (Euphorbiaceae)	Bacterial and fungal infections, including *S. Aureus, B. Cereus,* and *Candida albicans*	All the extracts exhibited broad-spectrum antibacterial activity, with the hexane extract of the yellow rootbark being the most active. It showed moderate antifungal activity against Candida albicans supporting its traditional use in treating different infections.
22	Akinpelu and Obuotor. 2000 [43]	Nigeria	Methanolic extract of the stem bark *Piliostigma thonningii* (Fabaceae)	Leprosy, smallpox, coughs, wounds, and ulcers	The extract *of Piliostigma thonningii* stem bark exhibited antibacterial activity against six out of eight tested bacteria at a concentration of 20 mg/ml.
23	Koné et al., 2004 [44]	Côte-d'Ivoire	67 crude ethanol extracts from leaves, stem barks, roots and whole plants of 50 plants belonging to Agavaceae, Amaranthaceae, Anacardiaceae, Annonaceae, Apocynaceae, Araliaceae, Asparagaceae, Bignoniaceae, Caesalpinaceae, Celastraceae, Chrysobalanaceae, Cochlospermaceae, Combretaceae, Convolvulaceae, Cyperaceae, Euphorbiaceae, Fabaceae, Hymenocardiaceae, Hyppocrateaceae, Loganiaceae, Malvaceae, Meliaceae, Mimosaceae, Moraceae, Olacaceae, Opiliaceae, Polygalaceae, Rubiaceae, Sterculiaceae, Verbenaceae and Vitaceae families	Abdominal pain, boils, conjunctivitis, constipation, cough, dermatitis, diarrhoea, dysentery, wounds, eyes infections, fever, gonorrhoea, heart problems, infected eruption intestinal worms, malaria, mouth wounds, respiratory diseases, rheumatism, sore throat, stomachache, toothache, vomiting	31 of the 67 extracts showed promising antibacterial activity against both Gram negative (*Escherichia coli* and *Pseudomonas aeruginosa*) and Gram positive (*Staphylococcus aureus, Enterococcus faecalis, Streptococcus pyogenes* and *Bacillus subtilis*) bacteria. 10 extracts derived from 10 plants belonging to 10 families showed considerable antibacterial activity. The families are Fabaceae, Caesalpinaceae, Sterculiaceae, Euphorbiaceae, Olacaceae, Meliaceae, Anacardiaceae, Vitaceae, Rubiaceae, Moraceae.
24	Asante-Kwatia et al., 2023 [45]	Ghana	*Ximenia americana (*Olacaceae)*, Justicia flava (*Acanthaceae)*, Jathropha multifida (*Euphorbiaceae)*, Bridelia tenufolia (*Zingiberaceae)*, Xylia evansii (*Leguminoceae)*, Anopyxis klaineana (*Rhizophoraceae)*, Withania somnifera (*Solanacea)*, Zanthoxylum zanthoxyliodes (*Rutaceae)*, Loranthus lecardi (*Loranthaceae)	S. mansoni Cercariae	All extracts exhibited a concentration and time-dependent cercaricidal activity against *S. mansoni* cercariae. Among all extracts tested*, B. tenufolia* and Z. *zanthoxyloides* exhibited the least cercaricidal activity.
25	Hudson et al., 2000 [46]	Togo	Antiviral activities of 10 species of Togolese medicinal plants that possess activity against herpes simplex virus. The species belong to the following family: Asteraceae, Bombacaceae,	Herpes simplex virus	The dominant activity of the plants was virucidal, although *Adansonia digitata* extracts appeared to also have intracellular antiviral activities. The anti-herpes simplex virus activity of the seven most active extracts was considerably enhanced by long wavelength ultraviolent light. All the extracts contained

			Commelinaceae, Davalliaceae, Malvacae, Moraceae, Rubiaceae, Rutaceae, Simarubaceae, Sapindaceae		antiviral photosensitizers, and the root-bark and leaf extracts of *Adansonia digitata* were the most potent.
26	Oyelami et al., 2003 [48]	Nigeria	*Acalypha wilkesiana* ointment (Euphorbiaceae)	Tinea pedis, Pityriasis versicolor, and Candida intetrigo	Acalypha ointment was very efficacious in treating these superficial fungal skin diseases, with a cure rate of 100% in each condition
27	Irobi et al., 2000 [49]	Nigeria	*Aspergillus quadrilineatus* (Aspergillaceae) extracts isolated from a Nigerian cereal	Antibiotic activity of extracts on fungal and bacterial species of clinical importance	*Aspergillus quadrilineatus* extracts demonstrated antibiotic activity against both fungal and bacterial species, indicating their potential as antimicrobial substances
28	Ankrah et al., 2003 [50]	Ghana	Plant decoction called AM-1 was formulated from *Jatropha curcas* (Euphorbiaceae)*, Gossypium hirsutum* (Mallows)*, Physalis angulate* (Solanaceae) *and Delonix regia* (Legumes)	Malaria	The AM-1 decoction eliminated malaria parasites (*Plasmodium falciparum* and *Plasmodium malarie*) from the peripheral blood of patients with malaria and did not show any undesired effects in patients or laboratory rats
29	Baba-Moussa et al., 1999 [51]	Northern regions of Benin and Togo	Antifungal activities of seven species of Combretaceae commonly used in traditional medicine in West Africa	Candida albicans, *Epidermophyton floccosum*, *Microsporum gypseum*, *Trichophyton mentagrophytes*, and *Trichophyton rubrum*	*Pteleopsis suberosa* and *Terminalia aicennioides* were the most active plants, and their antifungal activity may be attributed to their high content of tannins and saponins
30	Fleischer et al., 2002 [52]	Ghana	The leaves and flowering tops of *Acanthospermum hispidum (*Daisy)	Wide range of pathogenic bacteria	Ethanolic extracts of the leaves and flowering tops of *Acanthospermum hispidum* showed varying degrees of activity against a wide range of pathogenic bacteria, particularly Gram-positive organisms. The activity was mainly observed in the polar fractions of the alcoholic extract, while no activity was observed for the aqueous extract of the fresh plant material
31	Ebi and Ofoefule, 2003 [53]	Nigeria	The leaves of *Pterocarpu*s *osun (*Leguminoceae)	Five multidrug resistant *Escherichia coli* strains obtained from wounds	Methanol, ethanol, and aqueous leaves extracts of *Pterocarpu*s *osun* showed different zones of inhibition to the *Escherichia coli* isolates. Extracts of *Pterocarpu*s had broad spectrum of activity on the tested bacteria.
32	Konning et al., 2003 [54]	Ghana	*Aframomum melegueta (*Zingiberaceae), *Piper guineense (*Piperaceae)*, Xylopia aethiopica (*Annonaceae), and *Zingiber officinale (*Zingiberaceae)	Cough, bronchitis, rheumatism, dysenteric conditions, and skin infectious diseases	The methanol extracts of the plants showed significant antimicrobial activity against both Gram-positive and Gram-negative bacteria, as well as fungi

33	Okpekon et al., 2004 [55]	Cote d-Ivoire	Extracts from the leaves, roots, stem barks, trunk barks and whole plants of 17 herbal medicinal plants from 13 families namely: Annonaceae, Apocyanaceae, Combretaceae, Euphorbiaceae, Lythraceae, Meliaceae, Opiliaceae, Papilionaceae, Passifloraceae, Rubiaceae, Sapindaceae, Verbenaceae, Zingiberaceae.	Dysentery, helminthiasis, Hypertension, insect bites, jaundice, malaria, scabies, trypanosomiasis	*Anogeissus leiocarpus* and *Terminalia glaucescens*, showed strong activity against *Plasmodium falciparum*. *Lawsonia inermis* was selectively prescribed against trypanosomiasis and showed interesting trypanocidal activities Anthelmintic activities were found for 10 active species, and *Uvaria afzelli* and *Monodora myristica* were active against mites
34	Taiwo and Bolanle, 2003 [56]	Nigeria	The leaf essential oil of *Costus afer* (Costaceae)	Rheumatism, eye infections, headache, cough, diuretic, urethral discharge, and malaria	The essential oil of *Costus afer* from Nigeria contains 27 compounds, with sesquilavandulyl acetate being the principal component. However, the essential oil showed no antimicrobial activity
35	Traore-Keita et al., 2000 [57]	Mali	Aqueous, hydromethanol, and chloroform extractions of *Mitragyna inermis* (Rubiaceae)	Periodic fevers and malaria	The alkaloids contained in chloroform extracts and ursolic acid purified from the hydromethanol extract showed significant antimalarial activity, while the aqueous extracts did not show high antimalarial activity.
36	Anani et al., 2000 [59]	Togo	Methanol extracts of the leaf, root, bark, or whole plant of 19 medicinal plants used in Togo beloinging to the following family: Apocynaceae, Asteraceae, Bignoniaceae, Bombacaceae, Commelinaceae, Davalliaceae, Malvaceae, Moraceae, Orchidaceae, Rubiaceae, Rutaceae, Sapindaceae, Simarubaceae, Verbenaceae	*Staphylococcus aureus, Streptococcus fecalis, Bacillus subtilis, herpes simplex, Escherichia coli, Klebsiella pneumoniae, Salmonella typhimurium, Sindbis, Pseudomonas aeruginosa, poliovirus, Mycobacterium phlei, Candida albicans,*	Extracts showed significant antiviral activity, and all but two displayed antibiotic activity. Three species from the plant families Malvaceae, Asteraceae, Commelinaceae, were active against all three test viruses. Seven plants showed activity against only one or two viruses. The antibiotic profiles varied considerably, suggesting the presence of different bioactive phytochemicals in each species.

Discussion

This scoping review maps herbal remedies used for medicinal purposes in humans in West Africa. The findings indicate that the study of traditional medicines had continued and increased over time, thereby underscoring the enduring relevance of traditional medicine research, with contributions consistently observed over the years. There has also been an extensive exploration of medicinal plants across West African countries, including its use for the management of a range of diseases and disorders. The geographic distribution of these studies emphasizes the regional relevance of traditional medicine in addressing diverse health challenges. However, the number of countries in West Africa publishing on experimentations on traditional remedies, especially clinical trials, and ethnobotanical and ethnopharmacological surveys of plants used for traditional medicine is low, with research skewed towards Nigeria and Ghana.

One of the strengths of this study lies in its thorough examination of research pertaining to traditional medicine used in managing diseases in West Africa—a continent widely recognized for its considerable reliance on traditional healthcare practices for the well-being of its population [60]. However, the study had some limitations. First is the inclusion of only publications available and accessible in English, and second is limit of the search to three databases employed. These may have inadvertently excluded some research publications on traditional medicines use on West Africa since researchers in the sub-region also publish in French and other languages. Despite this limitation, the present study provides valuable insights into the subject matter.

First, there is diverse study designs employed in investigating traditional medicine in the region. The studies were majorly experimental in design, with a focus on laboratory-based

investigation of identified traditional remedies. In addition, a few clinical trials, representing systematic investigations on human participants, were conducted to evaluate the safety and efficacy of some traditional medical treatments. There are, however, the need for a lot more experimental and clinical trial studies that can facilitate the translation of the traditional medicines found to have therapeutic effects into commercial products as the validation of indigenous drugs is relevant to modern societies at large and helps to sustain local health care practices [61].

Furthermore, ethnobotanical and ethnopharmacological studies provided insights into the traditional knowledge, practices, and uses of plants, contributing valuable cultural context to the exploration. In addition, the ethnobotanical and ethnopharmacological surveys provide a deeper understanding of the traditional knowledge embedded in local communities. The surveys conducted in West Africa spotlighted a diverse array of plant families, plant parts, and preparation methods utilized in traditional remedies. Despite this wealth of knowledge, there remains untapped potential for further exploration using research methods tailored to the practice of traditional medicines [62]. These insights can be enhanced through dedicated studies focusing on specific diseases and their traditional medical remedies, as undertaken by those publications on schistosomiasis [45], cancer [ref], sickle cell [14], malaria [22, 29, 40, 50], and Herpes simplex virus [46]. Conducting disease-centric studies has the potential to aid the sub-region in developing therapeutic measures for disease entities categorized as neglected tropical diseases [63], which are of interest to the sub-region and currently receive limited global attention and funding.

The current study highlights the diverse therapeutic applications of identified traditional remedies, encompassing the management of symptomatic conditions and the targeted

treatment of specific diseases. However, the efficacies of these therapies remain inadequately studied, with only two identified clinical trials [48, 50]. To address this gap, countries should actively promote research on traditional medicines by formulating policies, guidelines, and providing funding for such endeavors. The development of policies and guidelines aimed at facilitating the seamless integration of traditional medicines into mainstream healthcare can play a pivotal role in encouraging and facilitating clinical trials in this field.

Nigeria [64, 65] and Ghana [66] are at the forefront of these integration efforts in the region. These pioneering initiatives not only aim to bridge the gap between traditional and modern medical practices but also foster a more comprehensive approach to healthcare that embraces the rich heritage of traditional healing methods. The progress made by these nations in the number of publications on traditional medicines reflect a dedicated commitment to exploring the potential benefits of traditional medicine within the broader healthcare framework. As these integration efforts unfold, they hold the promise of expanding therapeutic options for patients, fostering collaborative healthcare practices, and preserving the invaluable traditional knowledge passed down through generations. There are also ongoing efforts in Sierra Leone led by the World Health Organisation, to regulation the practice of traditional medicines in the country. The observed regional disparity in the countries contributing information on the use of plants for traditional medicines emphasizes the need for broader engagement and collaboration to enrich the diversity of perspectives and practices captured in research.

Frequently referenced plant families in medicinal studies in West Africa include Euphorbiaceae, Rubiaceae, Combretaceae, and Meliaceae. Euphorbiaceae has gained global recognition for its effectiveness in treating gastrointestinal disorders, respiratory complaints, skin problems, and inflammation and injuries [67]. Within the Rubiaceae family, plant

species predominantly found in warmer climates are indicated for the treatment of diverse conditions such as malaria, hepatitis, eczema, edema, cough, hypertension, diabetes, and sexual dysfunction. Biological screenings of plants used by traditional healers have demonstrated various beneficial activities, including antimalarial, antimicrobial, antihypertensive, antidiabetic, antioxidant, and anti-inflammatory properties [68].

Combretaceae species find extensive use in traditional medicine to address inflammation, infections, diabetes, malaria, bleeding, diarrhea, digestive disorders, and are employed as diuretics [69]. In the Meliaceae family, while various species are utilized for purposes such as vegetable oil, soap-making, and insecticides, Trichilia emetica, a plant native to Africa, holds significance in traditional medicine for treating a range of ailments such as abdominal pains, dermatitis, hemorrhoids, jaundice, and chest pain [70].

This scoping review highlights the need for broader regional engagement and collaboration on traditional medicines' use to capture diverse perspectives and its practices in West Africa. There is a pressing need for more experimental and clinical trial studies to validate indigenous medicines use and facilitate their translation into commercial products. This validation is crucial not only for the local healthcare practices but also improves access of populations in the sub-region to medical care and scale up possible management of diseases of concern to the sub-region.

In conclusion, this scoping review mapping the modalities employed in traditional medicine research reveals a rich tapestry of temporal, geographical, and methodological diversity. The findings underscore the enduring relevance of traditional medicine across West Africa and emphasize the need for continued research to harness its full potential for healthcare. The

study also highlights the need for further reviews of publications on traditional medicines written in other languages in the region in order to develop a rich compendium of the opportunities there are to harness the potential healthcare opportunities traditional medicines offer in the sub-region.

References

1. Abdullahi AA. Trends and challenges of traditional medicine in Africa. Afr J Tradit Complement Altern Med. 2011;8(5 Suppl):115-23. doi: 10.4314/ajtcam.v8i5S.5.
2. Feierman S. Struggles for Control: The Social Roots of Health and Healing in Modern Africa. African Studies Review. 1985; 28(2/3): 73-147.
3. Ezekwesili-Ofili JO, Okaka ANC. Herbal Medicines in African Traditional Medicine. 2019: DOI: 10.5772/intechopen.80348.
4. Sato A. Revealing the popularity of traditional medicine in light of multiple recourses and outcome measurements from a user's perspective in Ghana, Health Policy and Planning. 2012; 27(8): 625–637. https://doi.org/10.1093/heapol/czs010.
5. Putsch RW III, Joyce M. Dealing with Patients from Other Cultures. In: Walker HK, Hall WD, Hurst JW (editors). Clinical Methods: The History, Physical, and Laboratory Examinations (3rd edition). Boston: Butterworths; 1990.
6. World Health Organisation. Traditional medicine has a long history of contributing to conventional medicine and continues to hold promise.10 August 2023. Available at: https://www.who.int/news-room/feature-stories/detail/traditional-medicine-has-a-long-history-of-contributing-to-conventional-medicine-and-continues-to-hold-promise. Accessed: 18 November 2023.

7. Krah E, de Kruijf J, Ragno L. Integrating Traditional Healers into the Health Care System: Challenges and Opportunities in Rural Northern Ghana. J Community Health. 2018 Feb;43(1):157-163. doi: 10.1007/s10900-017-0398-4.
8. Kayombo EJ, Uiso FC, Mbwambo ZH, Mahunnah RL, Moshi MJ, Mgonda YH. Experience of initiating collaboration of traditional healers in managing HIV and AIDS in Tanzania. J Ethnobiol Ethnomed. 2007 Jan 26;3:6. doi: 10.1186/1746-4269-3-6.
9. Frass M, Strassl RP, Friehs H, Müllner M, Kundi M, Kaye AD. Use and acceptance of complementary and alternative medicine among the general population and medical personnel: a systematic review. Ochsner J. 2012 Spring;12(1):45-56.
10. Bulia, F. (2023). African Herbal Remedies: Traditional Healing Plants and Their Modern Uses. [online] POLISH DISTRIBUTOR OF RAW MATERIALS. Available at: https://naturalpoland.com/en/artykuly/produkty-z-afryki/african-herbal-remedies-traditional-healing-plants-and-their-modern-uses/ [Accessed 22 Dec. 2023].
11. Peters, M. D. J.; Marnie, C.; Tricco, A. C.; Pollock, D.; Munn, Z.; Alexander, L.; McInerney, P.; Godfrey, C. M.; Khalil, H. Updated Methodological Guidance for the Conduct of Scoping Reviews. JBI Evidence Synthesis 2020, 18 (10), 2119–2126. https://doi.org/10.11124/jbies-20-00167.
12. Tricco, A. C.; Lillie, E.; Zarin, W.; O'Brien, K. K.; Colquhoun, H.; Levac, D.; Moher, D.; Peters, M. D. J.; Horsley, T.; Weeks, L.; Hempel, S.; Akl, E. A.; Chang, C.; McGowan, J.; Stewart, L.; Hartling, L.; Aldcroft, A.; Wilson, M. G.; Garritty, C.; Lewin, S. PRISMA Extension for Scoping Reviews (PRISMA-ScR): Checklist and Explanation. Annals of Internal Medicine 2018, 169 (7), 467–473. https://doi.org/10.7326/m18-0850.
13. Prismastatement.org. https://www.prismastatement.org/Extensions/ScopingReviews?AspxAutoDetectCookieSupport=1. (accessed: 30 September 2023).

14. Mgbemene, C. N., & Ohiri, F. C. (1999). Anti-sickling Potential of Terminalia catappa Leaf Extract. Pharmaceutical Biology, 37(2), 152–154. https://doi.org/10.1076/phbi.37.2.152.6090
15. Diallo, D., Hveem, B., Mahmoud, M. E., Berge, G., Berit Smestad Paulsen, & Aboubacar Abdoulwahidou Maiga. (1999). An Ethnobotanical Survey of Herbal Drugs of Gourma District, Mali. Pharmaceutical Biology, 37(1), 80–91. https://doi.org/10.1076/phbi.37.1.80.6313
16. Nwosu, M. O. (1999). Herbs for mental disorders. Fitoterapia, 70(1), 58–63. https://doi.org/10.1016/s0367-326x(98)00024-0
17. Ilori, M. E., Sheteolu, A. O., Omonigbehin, E. A., & Adejuwon Adewale Adeneye. (1996). Antidiarrhoeal activities of Ocimum gratissimum (Lamiaceae). PubMed, 14(4), 283–285.
18. Alade, P. I., & Irobi, O. N. (1993). Antimicrobial activities of crude leaf extracts of Acalypha wilkesiana. Journal of Ethnopharmacology, 39(3), 171–174. https://doi.org/10.1016/0378-8741(93)90033-2
19. Koumaglo, K., Gbeassor, M., Nikabu, O., de Souza, C., & Werner, W. (1992). Effects of Three Compounds Extracted fromMorinda lucidaonPlasmodium falciparum. Planta Medica, 58(06), 533–534. https://doi.org/10.1055/s-2006-961543
20. Irobi, O. N., & Daramola, S. O. (1993). Antifungal activities of crude extracts of Mitracarpus villosus (Rubiaceae). Journal of Ethnopharmacology, 40(2), 137–140. https://doi.org/10.1016/0378-8741(93)90059-e
21. Irobi, O. N., & Banso, A. (1994). Effects of crude leaf extracts of Acalypha torta against some anaerobic bacteria. Journal of Ethnopharmacology, 43(1), 63–65. https://doi.org/10.1016/0378-8741(94)90118-x

22. Udeinya, I. J. (1993). Anti-malarial activity of Nigerian neem leaves. Transactions of the Royal Society of Tropical Medicine and Hygiene, 87(4), 471. https://doi.org/10.1016/0035-9203(93)90042-o
23. Olukoya, D. K., Idika, N., & Odugbemi, T. (1993). Antibacterial activity of some medicinal plants from Nigeria. Journal of Ethnopharmacology, 39(1), 69–72. https://doi.org/10.1016/0378-8741(93)90051-6
24. Olowo, S. F., Omotayo, A. O., Lawal, I. O., Ndhlovu, P. T., & Aremu, A. O. (2022). Ethnobotanical use-pattern for indigenous fruits and vegetables among selected communities in Ondo State, Nigeria. South African Journal of Botany, 145, 501-511.
25. Irobi, O. N. (1992). Activities of Chromolaena odorata (Compositae) leaf extract against Pseudomonas aeruginosa and Streptococcus faecalis. Journal of Ethnopharmacology, 37(1), 81–83. https://doi.org/10.1016/0378-8741(92)90007-e
26. Adomi PO. (2008). Screening of the leaves of three Nigerian medicinal plants for antibacterial activity. 7(15). https://doi.org/10.4314/ajb.v7i15.59084
27. Boadu, A. A., & Asase, A. (2017). Documentation of Herbal Medicines Used for the Treatment and Management of Human Diseases by Some Communities in Southern Ghana. Evidence-Based Complementary and Alternative Medicine, 2017, 1–12. https://doi.org/10.1155/2017/3043061
28. Amoah, L. E., Kakaney, C., Kwansa-Bentum, B., & Kusi, K. A. (2015). Activity of Herbal Medicines on Plasmodium falciparum Gametocytes: Implications for Malaria Transmission in Ghana. PLOS ONE, 10(11), e0142587. https://doi.org/10.1371/journal.pone.0142587
29. Cudjoe, E., Dickson Donu, Okonu, R., Amponsah, J. A., & Linda Eva Amoah. (2020). The In Vitro Antiplasmodial Activities of Aqueous Extracts of Selected Ghanaian Herbal

Plants. Journal of Parasitology Research, 2020, 1–8. https://doi.org/10.1155/2020/5041919

30. David, O.M., Famurewa O, & Olawale, A. K. (2010). In vitro assessment of aqueous and ethanolic extracts of some Nigerian chewing sticks on bacteria associated with dental infections. African Journal of Microbiology Research, 4(19), 1949–1953. https://doi.org/10.5897/ajmr.9000393
31. Healy, M. A., Aslam, M., & Bamgboye, O. A. (1984). Traditional medicine and lead-containing preparations in Nigeria. Public Health, 98(1), 26–32. https://doi.org/10.1016/s0033-3506(84)80056-6
32. Macfoy, C. A., & Sama, A. M. (1983). Medicinal plants in pujehun district of sierra leone. Journal of Ethnopharmacology, 8(2), 215–223. https://doi.org/10.1016/0378-8741(83)90055-7
33. Odebiyi, O., & Sofowora, E. (1979). Antimicrobial Alkaloids from a Nigerian Chewing Stick (Fagara zanthoxyloides). Planta Medica, 36(07), 204–207. https://doi.org/10.1055/s-0028-1097271
34. Ogbole, O. O., Akinleye, T. E., Segun, P. A., Faleye, T. C., & Adeniji, A. J. (2018). In vitro antiviral activity of twenty-seven medicinal plant extracts from Southwest Nigeria against three serotypes of echoviruses. Virology Journal, 15(1). https://doi.org/10.1186/s12985-018-1022-7
35. Rotimi, V. O., & Mosadomi, H. A. (1987). The effect of crude extracts of nine African chewing sticks on oral anaerobes. Journal of Medical Microbiology, 23(1), 55–60. https://doi.org/10.1099/00222615-23-1-55
36. Sonibare, M. A., Moody, J. O., & Adesanya, E. O. (2009). Use of medicinal plants for the treatment of measles in Nigeria. 122(2), 268–272. https://doi.org/10.1016/j.jep.2009.01.004

37. Grønhaug, T. E., Glæserud, S., Skogsrud, M., Ballo, N., Bah, S., Diallo, D., & Paulsen, B. S. (2008). Ethnopharmacological survey of six medicinal plants from Mali, West-Africa. Journal of Ethnobiology and Ethnomedicine, 4(1). https://doi.org/10.1186/1746-4269-4-26

38. Adesina, S. K., Idowu, O., Ogundaini, A. O., Oladimeji, H., Olugbade, T. A., Onawunmi, G. O., & Pais, M. (2000). Antimicrobial constituents of the leaves ofAcalypha wilkesiana and Acalypha hispida. Phytotherapy Research, 14(5), 371–374. https://doi.org/10.1002/1099-1573(200008)14:5%3C371::aid-ptr625%3E3.0.co;2-f

39. Agyare, C., Spiegler, V., Asase, A., Scholz, M., Hempel, G., & Hensel, A. (2018). An ethnopharmacological survey of medicinal plants traditionally used for cancer treatment in the Ashanti region, Ghana. Journal of Ethnopharmacology, 212, 137–152. https://doi.org/10.1016/j.jep.2017.10.019

40. Köhler, I., Jenett-Siems, K., Kraft, C., Siems, K., Abbiw, D., Bienzle, U., & Eich, E. (2002). Herbal Remedies Traditionally Used Against Malaria in Ghana: Bioassay-Guided Fractionation of Microglossa pyrifolia (Asteraceae). Zeitschrift Für Naturforschung C, 57(11-12), 1022–1027. https://doi.org/10.1515/znc-2002-11-1212

41. Ajali, U. (2000). Antibacterial activity of Alchornea cordifolia stem bark. Fitoterapia, 71(4), 436–438. https://doi.org/10.1016/s0367-326x(00)00131-3

42. Aiyelaagbe, O. O., Adesogan, E. K., Ekundayo, O., & Adeniyi, B. A. (2000). The antimicrobial activity of roots ofJatropha podagrica (Hook). Phytotherapy Research, 14(1), 60–62. https://doi.org/10.1002/(sici)1099-1573(200002)14:1%3C60::aid-ptr597%3E3.0.co;2-b

43. Akinpelu, D. A., & Obuotor, E. M. (2000). Antibacterial activity of Piliostigma thonningii stem bark. Fitoterapia, 71(4), 442–443. https://doi.org/10.1016/s0367-326x(00)00136-2

44. Koné, W. M., Atindehou, K. K., Terreaux, C., Hostettmann, K. U. R. T., Traore, D., & Dosso, M. (2004). Traditional medicine in North Côte-d'Ivoire: screening of 50 medicinal plants for antibacterial activity. Journal of ethnopharmacology, 93(1), 43-49.
45. Asante-Kwatia, E., Gyimah, L., Arnold Donkor Forkuo, Anyan, W. K., Makafui Adzo Gbemu, Francis Ackah Armah, & Abraham Yeboah Mensah. (2023). Ethnobotanical Survey and Cercaricidal Activity Screening of Medicinal Plants Used for Schistosomiasis Treatment in Atwima-Nwabiagya District, Ashanti Region, Ghana. Journal of Parasitology Research, 2023, 1–15. https://doi.org/10.1155/2023/6707157
46. Hudson, J. B., Anani, K., Lee, M. K., de Souza, C., Arnason, J. T., & Gbeassor, M. (2000). Further Investigations on the Antiviral Activities of Medicinal Plants of Togo. Pharmaceutical Biology, 38(1), 46–50. https://doi.org/10.1076/1388-0209(200001)3811-bft046
47. Togola, A., Diallo, D., Dembélé, S., Barsett, H., & Paulsen, B. S. (2005). Ethnopharmacological survey of different uses of seven medicinal plants from Mali, (West Africa) in the regions Doila, Kolokani and Siby. Journal of Ethnobiology and Ethnomedicine, 1(1). https://doi.org/10.1186/1746-4269-1-7
48. Oyelami, O.A., Onayemi, O., Oladimeji, F.A., Ogundaini, A. O., Olugbade, T. A., & Onawunmi, G. O. (2003). Clinical evaluation ofAcalypha ointment in the treatment of superficial fungal skin diseases. Phytotherapy Research, 17(5), 555–557. https://doi.org/10.1002/ptr.1161
49. Irobi, O. N., Gbodi, T. A., Moo-Young, M., & Anderson, W. A. (2000). Antibiotic Activity of Aspergillus quadrilineatus Extracts Isolated From a Nigerian Cereal. Pharmaceutical Biology, 38(1), 57–60. https://doi.org/10.1076/1388-0209(200001)3811-bft057

50. Ankrah, N.-A., Nyarko, A. K., Addo, P. G. A., Ofosuhene, M., Dzokoto, C., Marley, E., Addae, M. M., & Ekuban, F. A. (2003). Evaluation of efficacy and safety of a herbal medicine used for the treatment of malaria. Phytotherapy Research, 17(6), 697–701. https://doi.org/10.1002/ptr.1196

51. Baba-Moussa, F., Akpagana, K., & Bouchet, P. (1999). Antifungal activities of seven West African Combretaceae used in traditional medicine. Journal of Ethnopharmacology, 66(3), 335–338. https://doi.org/10.1016/s0378-8741(98)00184-6

52. Fleischer, T. C., Ameade, E. P. K., & Sawer, I. K. (2003). Antimicrobial activity of the leaves and flowering tops of Acanthospermum hispidum. Fitoterapia, 74(1-2), 130–132. https://doi.org/10.1016/s0367-326x(02)00290-3

53. Ebi, G. C., & Ofoefule, S. I. (2000). Antimicrobial activity of Pterocarpus osun stems. Fitoterapia, 71(4), 433–435. https://doi.org/10.1016/s0367-326x(00)00130-1

54. Konning, G. H., Agyare, C., & Ennison, B. (2004). Antimicrobial activity of some medicinal plants from Ghana. Fitoterapia, 75(1), 65–67. https://doi.org/10.1016/j.fitote.2003.07.001

55. Okpekon, T., Yolou, S., Gleye, C., Roblot, F., Loiseau, P., Bories, C., Grellier, P., Frappier, F., Laurens, A., & Hocquemiller, R. (2004). Antiparasitic activities of medicinal plants used in Ivory Coast. Journal of Ethnopharmacology, 90(1), 91–97. https://doi.org/10.1016/j.jep.2003.09.029

56. Taiwo, A. O., & Bolanle, A. A. (2003). The leaf essential oil of Costus afer Ker-Grawl from Nigeria. Flavour and Fragrance Journal, 18(4), 309–311. https://doi.org/10.1002/ffj.1186

57. Traore-Keita, F., Gasquet, M., Di Giorgio, C., Ollivier, E., Delmas, F., Keita, A., Doumbo, O., Balansard, G., & Timon-David, P. (2000). Antimalarial activity of four

plants used in traditional medicine in Mali. Phytotherapy Research, 14(1), 45–47. https://doi.org/10.1002/(sici)1099-1573(200002)14:1%3C45::aid-ptr544%3E3.0.co;2-c

58. Catarino, L., Havik, P. J., & Romeiras, M. M. (2016). Medicinal plants of Guinea-Bissau: Therapeutic applications, ethnic diversity and knowledge transfer. Journal of Ethnopharmacology, 183, 71–94. https://doi.org/10.1016/j.jep.2016.02.032.
59. Anani K, Hudson JB, de Souza C, Akpagana K, Tower GHN, Arnason JT, Gbeassor M. Investigation of Medicinal Plants of Togo for Antiviral and Antimicrobial Activities, Pharmaceutical Biology. 2000; 38:1, 40-45, DOI: 10.1076/1388-0209(200001)3811-BFT040.
60. James, P.B., Wardle, J., Steel, A. and Adams, J. (2018). Traditional, complementary and alternative medicine use in Sub-Saharan Africa: a systematic review. BMJ Global Health, 3(5), pp.1–18. doi:https://doi.org/10.1136/bmjgh-2018-000895.
61. Leonti M, Casu L. Traditional medicines and globalization: current and future perspectives in ethnopharmacology. Front Pharmacol. 2013 Jul 25;4:92. doi: 10.3389/fphar.2013.00092.
62. Liu JP, Chen KJ; guideline development team. Methodology guideline for clinical studies investigating traditional Chinese medicine and integrative medicine: executive summary. Complement Ther Med. 2015 Oct;23(5):751-6. doi: 10.1016/j.ctim.2015.08.001.
63. https://www.who.int/news-room/questions-and-answers/item/neglected-tropical-diseases.
64. Federal Ministry of Health. Traditional Medicine Policy for Nigeria. 2007.
65. Osuide GE. Chapter 21 - Regulation of herbal medicines in Nigeria: the role of the National Agency for Food and Drug Administration and Control (NAFDAC). Maurice M Iwu, Jacqueline C Wootton (Eds). Advances in Phytomedicine, Elsevier. 2002: 249-258. https://doi.org/10.1016/S1572-557X(02)80030-7.

66. World Health Organisation. Traditional healers broaden health care in Ghana. 12 December 2019. Available at: https://www.afro.who.int/photo-story/traditional-healers-broaden-health-care-ghana. Accessed 22 December 2023.
67. Ernst M, Grace OM, Saslis-Lagoudakis CH, Nilsson N, Simonsen HT, Rønsted N. Global medicinal uses of Euphorbia L. (Euphorbiaceae). J Ethnopharmacol. 2015 Dec 24;176:90-101. doi: 10.1016/j.jep.2015.10.025.
68. Karou SD, Tchacondo T, Ilboudo DP, Simpore J. Sub-Saharan Rubiaceae: a review of their traditional uses, phytochemistry and biological activities. Pak J Biol Sci. 2011 Feb 1;14(3):149-69. doi: 10.3923/pjbs.2011.149.169.
69. de Morais Lima GR, de Sales IR, Caldas Filho MR, de Jesus NZ, de Sousa Falcão H, Barbosa-Filho JM, Cabral AG, Souto AL, Tavares JF, Batista LM. Bioactivities of the genus Combretum (Combretaceae): a review. Molecules. 2012 Aug 2;17(8):9142-206. doi: 10.3390/molecules17089142.
70. Komane BM, Olivier EI, Viljoen AM. Trichilia emetica (Meliaceae) – A review of traditional uses, biological activities and phytochemistry. Phytochemistry Letters. 2011; 4(1): 1-9. https://doi.org/10.1016/j.phytol.2010.11.002.

Appendix 1: Search Strategy

Total:6948

Duplicate:540

CINAHL complete=70

S/N	Keywords	Search Terms
1	Traditional medicine	TI (traditional medicine or complementary and alternative medicine or herb medicine) OR TI (herbal medicine or herbalism or herbal supplements) OR TI medicinal plant extracts OR TI african traditional medicine OR TI indigenous medicine OR TI Ethnomedicine
2	Africa	TI Africa OR TI (Saharan africa or sub-saharan africa or Sahara or sub-sahara or ssa)

Web of Science= 2768

S/N	Keywords	Search Terms
1	Traditional medicine	((((((((TS=(Traditional medicine)) OR TS=(Herbal medicine)) OR TS=(Medicinal plant)) OR TS=(Plant extract)) OR TS=(Herbal drugs)) OR TS=(African Traditional medicine)) OR TS=(Indigenous Medicine)) OR TS=(Home Remedies)) OR TS=(Ethnomedicine) and Preprint Citation Index (Exclude – Database)
2	West Africa	((((((((((((((((TS=(west africa)) OR TS=(Benin)) OR TS=(Burkina Faso)) OR TS=(Cape Verde)) OR TS=(Côte D'Ivoire)) OR TS=(Gambia)) OR TS=(Ghana)) OR TS=(Guinea)) OR TS=(Guinea-Bissau)) OR TS=(Liberia)) OR TS=(Mali)) OR TS=(Mauritania)) OR TS=(Niger)) OR TS=(Nigeria)) OR TS=(Senegal)) OR TS=(Sierra Leone)) OR TS=(Togo) and Preprint Citation Index (Exclude – Database)

PubMed= 646

	Keywords	Search strategy
#1	Traditional Medicine	((((((((Herbal medicine[Title/Abstract]) OR (Traditional medicine[Title/Abstract])) OR (Medicinal plant[Title/Abstract])) OR (Plant extract[Title/Abstract])) OR (Herbal drugs[Title/Abstract])) OR (African Traditional medicine[Title/Abstract])) OR (Indigenous Medicine[Title/Abstract])) OR (Home Remedies[Title/Abstract])) OR (Ethnomedicine[Title/Abstract])
#2	Use	“use"[Title/Abstract]
#3	Africa	“Africa"[Title/Abstract]
#4	#1 AND #2 AND #3	

Appendix 2: A compendium of plant families used for medicinal purposes in West Africa

1. Acanthaceae [24, 27, 36, 39, 45, 48]
2. Aizoaceae [15]
3. Aloaceae [27]
4. Amaranthaceae [16, 24, 27, 32, 39]
5. Amaryllidaceae [39]
6. Anacardiaceae [24, 32, 39, 58]
7. Anisophylleaceae [58]
8. Annonaceae [27, 39, 45, 58]
9. Anonaceae [32]
10. Apiaceae [15]
11. Apocynaceae [27, 39, 45, 58]
12. Araceae [32, 39]
13. Araliaceae [39]
14. Arecaceae [15, 24, 39, 658]
15. Asclepiadaceae [15, 277, 32, 36]
16. Asparagaceae [58]
17. Asteraceae [24, 27, 36, 39, 45, 58]
18. Balanitaceae [15]
19. Basellaceae [24]
20. Bignoniaceae [16, 27, 36, 58]
21. Bombacaceae [15, 27]
22. Boraginaceae [27, 39, 47]
23. Bromeliaceae [39]
24. Burseraceae [15, 39]
25. Caesalpiniaceae [32, 47]
26. Calophyllaceae [39]
27. Cannabaceae [58]
28. Capparaceae [15, 58]
29. Capparidaceae [27]
30. Caricaceae [32, 39, 58],
31. Casuarinaceae [39]
32. Celastraceae [15, 32, 39, 58]
33. Chrysobalanaceae [27, 58]
34. Cleomaceae [39]
35. Clusiaceae [39]
36. Cochlospermaceae [47]
37. Combretaceae [15, 27, 37, 39, 58]
38. Compositae [32]
39. Connaraceae [32, 39, 58]
40. Convolculaceae [32, 56]
41. Crassulaceae [24, 32, 39]
42. Crassulaceae [39]
43. Cucurbetaciae [15, 27, 36, 39, 45, 59]
44. Cyperaceae [15, 27, 39, 58]
45. Dennstaedtiaceae [27]
46. Dichapetalaceae [45]
47. Dilleniaceae [58]
48. Dioscoreaceae [36, 39, 58]
49. Ditleniaceae [32]
50. Ebenaceae [32, 58]
51. Euphorbiaceae [16, 24, 27,32, 39, 45, 58]
52. Fabaceae [15, 16, 24, 27, 39, 58]
53. Flacourtiaceae [32]
54. Gentianaceae [58]
55. Hypericaceae [32, 58]
56. Hypoxidaceae [58]
57. Icacinaceae [58]
58. Irvingiaceae [24],
59. Labiatae [32, 36]
60. Lamiaceae [24, 27, 39, 58]
61. Lauraceae [58]
62. Leguminosae [36, 37, 45, 47],
63. Liliaceae [16, 45]
64. Loganiaceae [16, 32, 47, 58]
65. Loranthaceae [36, 45, 58]
66. Malpighiaceae [58]
67. Malvaceae [15, 24, 27, 32, 39, 58]
68. Marantaceae [39]
69. Melastomataceae [32, 58]
70. Meliaceae [15, 16, 27, 32, 39, 45, 47, 58]
71. Menispermaceae [16, 27, 39, 58]
72. Mimosaceae [32]
73. Moraceae [24, 32]
74. Moraceae [39, 58]
75. Moringaceae [27, 58]
76. Musaceae [39],
77. Myristicaceae [16, 39]
78. Myrtaceae [16, 27, 39, 45]
79. Nyctaginaceae [16, 24, 39]
80. Nymphaeaceae [15],
81. Olacaceae [37, 45, 58]
82. Opiliaceae [37, 47, 58]
83. Orchidaceae [58]
84. Oxalidaceae [37]
85. Palmae [32, 36]
86. Pandaceae [38]
87. Papaveraceae [36, 39, 58]
88. Papilionaeeae [32, 47]
89. Passifloraceae [16, 32, 39, 58]
90. Pedaliaceae [32]
91. Phyllanthaceae [39, 58]
92. Piperaceae [36, 39, 58]
93. Plantaginaceae [58]
94. Poaceae [15, 36, 39, 58]
95. Polygalaceae [39, 58]
96. Portulaceae [32, 39]
97. Rhamnaceae [15 32]
98. Rhizophoraceae [45],
99. Rubiaceae [15, 16, 32, 39, 45, 58]
100. Rutaceae [24, 27, 32, 39, 45, 58]
101. Salicaceae [39]
102. Salvadoraceae [15]
103. Sapindaceae [24, 27, 36, 39, 58]
104. Sapotaceae [24, 27, 36, 58]
105. Selaginellaceae [32]
106. Simaroubaceae [58]
107. Smilacaceae [58]
108. Solanaceae [24, 27, 32, 36, 39, 45, 58]

109. Sterculiaceae [15, 37]
110. Talinaceae [24]
111. Thymelaeaceae [58]
112. Tiliaceae [15, 36]
113. Verbenaceae [16, 39, 58]
114. Vitaceae [58]
115. Zingiberaceae [16, 32, 36, 39, 45, 58]
116. Zygophyllaceae [39, 45]

Appendix 3: Compendium of plant families used for experimentation

1. Acanthaceae [45]
2. Agavaceae [44]
3. Amaranthaceae [40, 44. 29, 35]
4. Annonaceae [29, 34, 35, 44, 55, 56]
5. Apocynaceae [28, 40, 44, 55, 59]
6. Araliaceae [44]
7. Asclepiadaceae [40]
8. Asparagaceae [44]
9. Aspergillaceae [49]
10. Asteraceae [40]
11. Astraceae [46, 59]
12. Bignoniaceae [44, 59]
13. Bombacaceae [40, 46, 59]
14. Caesalpinaceae [44]
15. Celastraceae [44]
16. Chrysobalanaceae [44]
17. Cochlospermaceae [44]
18. Combretaceae [14, 23, 30, 35, 44, 51, 55]
19. Commelinaceae [46, 39]
20. Convolvulaceae [44]
21. Costaceae [56]
22. Cyperaceae [44]
23. Daisy [25, 35, 52]
24. Davalliaceae [46, 59]
25. Euphorbiaceae [18, 21, 29, 38, 39, 41, 42, 44, 45, 47, 50, 55],
26. Fabaceae [29, 35, 44]
27. Hymenocardiaceae [44]
28. Hyppocrateaceae [44]
29. Lamiaceae [17]
30. Legumes [50]
31. Leguminoceae [45, 53]
32. Loganiaceae [44]
33. Loranthaceae [45]
34. Lythraceae [55]
35. Madder [35]
36. Mahogany [22]
37. Mallows [50]
38. Malvacae [44, 46, 59]
39. Meliaceae [44, 55]
40. Mimosaceae [40, 44]
41. Moraceae [44, 46, 59]
42. Moringaceae [29]
43. Olacaceae [44, 45]
44. Opiliaceae [44, 55]
45. Orchidaceae [59]
46. Papilionaceae [55]
47. Passifloraceae [55]
48. Piperaceae [30]
49. Polygalaceae [44]
50. Rhizophoraceae [45]
51. Rubiaceae [19, 20, 26, 30, 35, 40, 44, 46, 55, 57, 59]
52. Rutaceae [30, 33, 35, 45, 46, 59]
53. Sapindaceae [46, 55, 59]
54. Sapotaceae [30, 35]
55. Simarubaceae [46, 59]
56. Solanacea [45, 50]
57. Sterculiaceae [44]
58. Verbenaceae [44, 55, 59]
59. Vitaceae [44]
60. Zingiberaceae [45, 54, 55]

Click here to access/download
Supporting Information
PRISMA-ScR-Fillable-Checklist_Trad.docx
