# Supplementary material for: A scoping review of the use of traditional medicine for the management of ailments in West Africa
Source: PLoS One. 2024 Jul 8;19(7):e0306594. doi: 10.1371/journal.pone.0306594 (PMC11230574; doi:10.1371/journal.pone.0306594)
Supplement: Attachment — Submitted filename: Review report.docx [file pone.0306594.s005.docx]

**Review report**

The manuscript titled “A scoping review on the use of traditional medicine for the management of ailments in West Africa” presented a comprehensive overview of traditional healing practices in West Africa. It is an important study as it adds knowledge to the field of complementary and alternative medicine. The data search was generally well executed and methods clear. However, there are major issues with especially the presentation of results; I can only recommend publication of the work in this journal when authors make substantial amendments to the manuscript in the different sections as per these recommendations.

**Title page**: match authors correctly to their affiliations; i do not see authors affiliated to 1, 2, 3.

**Abstract**: arrange words alphabetically

**Introduction**: correct the grammar in highlighted section

**Methods**: correct grammar in highlighted sentences; replace appendix with supplementary file here and throughout the manuscript; specify data analysis software used

**Results**: be consistent in using figures or words as highlighted in different sections; table 1- All scientific names of all plants and some pathogens in the table are incorrectly written-not italicized and in some cases species names are started in uppercase. Go through the entire table carefully and correct these; You have mixed hyphenated words (e.g. Anti-malarial) and non-hyphenated ones (e.g. Antimalarial) throughout the manuscript body and tables, please be consistent. Pay attention to all such words throughout the manuscript; your citation format is inconsistent; rephrase sentences as recommended; write correct plant family names; table 3- A number of plant species and pathogen names are not scientifically correct throughout the table. Be keen to spell correctly and italicize

**Discussion**: This is fairly written, although some parts are loose and very general. It can be concretized in context; rephrase ad reorganize as recommended; include missing intext citation; correct the grammar and cite source of sentences as pointed out.

**References**: Different reference formats were used. Refer to journal guidelines and carefully go through each of the references to harmonize.

**Recommendation**: Since it is a scoping review, I would like to recommend that authors investigate the existence of correlation of usage of various plant parts, plant families, preparation methods with geographical location or climate.
