# Supplementary material for: A scoping review of the use of traditional medicine for the management of ailments in West Africa
Source: PLoS One. 2024 Jul 8;19(7):e0306594. doi: 10.1371/journal.pone.0306594 (PMC11230574; doi:10.1371/journal.pone.0306594)
Supplement: Attachment — Submitted filename: REVIEW COMMENTS.docx [file pone.0306594.s008.docx]

Some efforts have been made by the authors to improve the quality of the manuscript based on the reviewers’ comments. However, there are still some grammatical errors in the document. I suggest the authors have the manuscript subjected to English editing and proofreading by institutions that offer these services.

Some of the issues that still need to be addressed by the authors in the different sections are here highlighted.

**Abstract:**

Key words must be arranged alphabetically

**Introduction:**

The sentence “This health-seeking behaviors across the region creating a nuanced healthcare ecosystem [6] and underscores the importance of acknowledging diverse healthcare practices and fostering collaborative efforts between traditional healers and modern healthcare professionals [7, 8].” is incorrect grammatically. Phrases used are pertinent to those generated by IA tools. Please rephrase and correct grammar.

**Methodology:**

In this sentence “The review was guided by the research questions: What is the extent and nature of Englishlanguage publications on the utilization of traditional medicine for treating various ailments in

West Africa.”

You talk of questions but I only see one.

In this sentence “A systematic search of the literature was conducted in PubMed, Web of Science, and the Cumulative Index of Nursing and Allied Health Literature (CINAHL) using the terms shown in S1 File.”

At first mention, write “S1 File” fully.

In this sentence “Thereafter, duplicate publications were removed. Three researchers (ORA, MTO, MOF) performed a screening of the titles and abstracts of the downloaded articles independently using pre-defined inclusion and exclusion criteria.”

write as “performed screening” and not” performed a screening”

In this sentence “Studies were included if there was an agreement between all the reviewers”

Use “among” instead of “between”. I don’t suppose the agreement was amongst reviewers but rather authors.

In this sentence “Animal studies were excluded. Also excluded were studies on non-African populations. Unpublished theses and dissertations, letters to the editor, commentaries on studies, scoping, systematic and narrative reviews, and studies whose full lengths cannot be accessed were excluded. Also, studies with insufficient results suitable for analysis were also excluded.”

Replace “cannot be accessed” with “could not be accessed”

In this sentence “As shown in Table 1, the publication period for the 49 manuscripts spanned from 1979 to 2023. The distribution across time revealed four (8.2%) were published between 1979 and 1990 [31- 33, 35], 20 (40.8%) were published between 1991 and 2000 [14-19, 20-23, 25, 38, 41-43, 46, 49, 51, 57, 59], 13 (26.5%) were from 2001 to 2010 [26, 36, 37, 40, 44, 47, 48, 50, 52-56], seven (14.3%) papers from 2011 to 2020 [27-30, 39, 58, 62], and five (10.2%) papers from 2021 to 2023 [24, 34, 45, 59, 61].”

you have mixed use of figures and words e.g. “revealed four (8.2%)” and “20 (40.8%) were published”. Please harmonize here and all through the results section.

**Results**

in table 1, there are a numbers of plant family names italicized. This is incorrect; please italicize only Genus and Species names. e.g. “Antidiarrhoeal Activities of Ocimum gratissimum (Lamiaceae)” do not italicize “Lamiaceae.” Do this throughout the document.

Throughout the texts in most cases family names have been italicized; please do not italicize. Do this throughout table 2.

**Discussion**

“Leaves, often rich in bioactive compounds, are frequently utilized for their therapeutic properties in various remedies and are prepared in a various forms ranging from decoctions to poultices. Roots are also valued for their medicinal properties and are often prepared as decoctions or infusions to extract their therapeutic properties. The range of plant parts used for medicinal purposes and the forms they are used in the current study reflects the diverse traditional knowledge and practices embedded in local communities across the West Africa”

Change “to extract their therapeutic properties” to “to extract their active ingredients”
